# Capitalizing on integrated landscape approaches for wildfire risk mitigation in the Mediterranean region

**DOI:** 10.1016/j.isci.2026.116741

**Published:** 2026-07-28

**Authors:** Elsa Varela, Mónica Hernández-Morcillo, Marion Jay, Davide Ascoli, Miltiadis Athanasiou, Valentina Bacciu, Maria João Canadas, Guillem Canaleta, Paula Castro, Iago Otero, Anabela Paula, Fernando Pulido, Núria Prat, Michele Salis, Fantina Tedim, Gavriil Xanthopoulos, Tobias Plieninger

**Affiliations:** 1 Social-Ecological Interactions in Agricultural Systems, University of Göttingen and University of Kassel, Göttingen 37073, Germany; 2 Institute of Public Goods and Policies, Spanish National Research Council (IPP-CSIC), Madrid 28037, Spain; 3 Eberswalde University for Sustainable Development, Eberswalde 16225, Germany; 4 IUCN Centre for Mediterranean Cooperation (IUCN Med), Málaga 29590, Spain; 5 Department of Agriculture, Forest and Food Sciences, University of Torino, Grugliasco 10095, Italy; 6 Institute of Mediterranean Forest Ecosystems, ELGO-DIMITRA, Athens 11528, Greece; 7 National Research Council, Institute of BioEconomy (CNR-IBE), Sassari 07100, Italy; 8 Forest Research Centre, TERRA Associate Laboratory, School of Agriculture, University of Lisbon, Lisbon 1349-017, Portugal; 9 Pau Costa Foundation, Taradell, Barcelona 08552, Spain; 10 Centre for Functional Ecology - Science for People and the Planet, Terra Associate Laboratory, Department of Life Sciences, University of Coimbra, Coimbra 3000-456, Portugal; 11 Interdisciplinary Centre for Mountain Research, University of Lausanne, CH-1967 Bramois (Sion), Lausanne, Valais, Switzerland; 12 INDEHESA - Instituto de Investigación de la Dehesa, Universidad de Extremadura, Plasencia 10600, Spain; 13 Centre of Studies in Geography and Spatial Planning (CEGOT), Faculty of Arts and Humanities, University of Porto, Porto 4150-564, Portugal

**Keywords:** landscape governance, fire-prone landscapes, fuel management strategies, landscape fire resilience, adaptive management

## Abstract

Wildfires in the Northern Mediterranean are increasing in frequency and intensity, driven by climate change, land abandonment, and fuel accumulation. Traditional fire suppression approaches are insufficient to address the complex social-ecological drivers of wildfire risk. This paper advocates adopting landscape approaches as an integrated and adaptive framework for mitigation. We assess ten wildfire mitigation initiatives in four countries, using five dimensions: multifunctionality, synergies and trade-offs, cross-sectoral integration, participation, and adaptive management. High-performing initiatives showed integrative planning, multi-actor governance, and adaptive learning. Key enablers of landscape coherence include strong leadership, institutional support, and participatory governance. However, gaps emerged in monitoring trade-offs and aligning strategies across sectors and spatial scales. Advancing landscape approaches in wildfire policy requires greater investment in collaboration, shared learning, and long-term governance capacities. Our findings offer insights to scale multifunctional, adaptive, and socially legitimate wildfire mitigation strategies in Mediterranean landscapes and beyond.

## Highlights

• Landscape approaches are proposed to integrate wildfire risk mitigation strategies
• Ten Mediterranean initiatives are assessed across five landscape approach dimensions
• Long-term governance, funding, and collaboration are essential for scaling up
• Trade-off monitoring and adaptive management are the weakest dimensions found

## Graphical abstract

**Figure:**
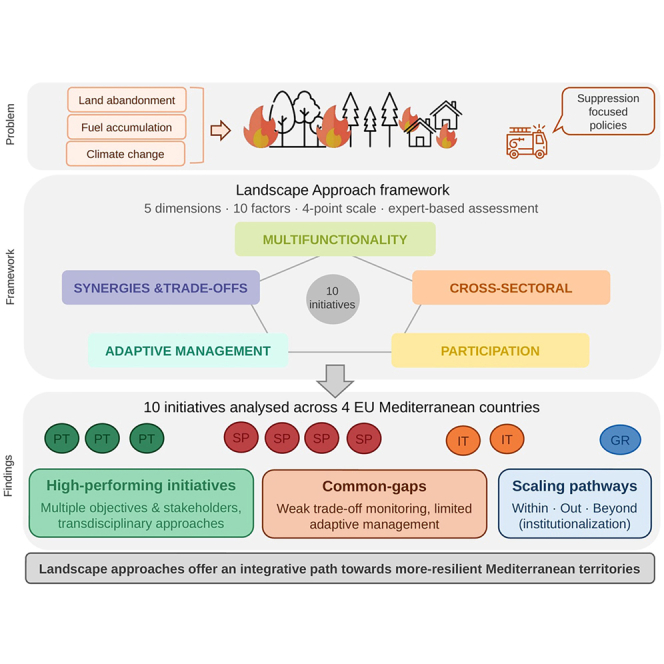

## Teaser

Weathering; Global change; Environmental event

## Introduction

Fire is a key natural process that has shaped many healthy ecosystems globally. However, wildfires are growing in frequency, intensity, and extent,^1^^,^^2^ becoming a major environmental and socioeconomic threat across many regions.^3^ Anthropogenic drivers such as land-use changes and carbon emissions result in fuel and climatic conditions that make wildfires more destructive.^2^ As extreme wildfires become more frequent, suppression costs^4^ and impacts on human values, assets, and ecosystem services increase.^3^^,^^5^ Increasingly less predictable fire regimes also overwhelm emergency response systems and can jeopardize the safety of firefighters, local communities, and ecosystems.^6^

So far, the dominant approach to firefighting in the Global North has been to suppress wildfires, including prohibiting fire use^7^ and mitigating and combating wildfires through structural (e.g., fuelbreaks) or technological (e.g., firefighting planes) interventions to eliminate or modify the hazard itself.^2^ Suppression can indeed support mitigation when both strategies are carefully integrated, for example by enabling fire spread under moderate conditions to reduce risk.^8^ However, the limits of fire suppression-centered strategies become evident in technologically well-equipped countries where many of these wildfires burn under extreme weather conditions and their behavior exceeds the limits of suppression.^8^ Furthermore, reactive suppression strategies often cause a “fire paradox” through biomass accumulation, creating favorable conditions for high-magnitude events with large burnt areas in years with adverse weather.^9^^,^^10^ These limitations exemplify the “wicked” nature of wildfire risk management^11^^,^^12^ that is^3^ shaped by the interdependency among forest management, civil protection policies, land-use planning, agriculture development, climate, and energy policies^13^ and that requires moving beyond sectoral approaches toward social-ecological strategies at landscape scale.^14^^,^^15^

The Northern Mediterranean region is a highly fire-prone area.^16^ Although fires are a natural element of Mediterranean-type forest ecosystems, large-scale wildfires are relatively new.^17^ Land abandonment and rural exodus triggered the replacement of traditional rural landscape mosaics by simplified, homogeneous landscapes that display favorable conditions for the spread of high-magnitude wildfires by connecting fuel loads available to burn.^18^^,^^19^^,^^20^^,^^21^^,^^22^^,^^23^ In the Northern Mediterranean region, many fire-prone territories are composed of forest patches intermingled with grazing and agricultural lands, urban areas, and industrial sites. This requires that whole landscapes are managed coherently for sustainable wildfire risk management,^24^ which includes the adoption of cross-cutting and transdisciplinary approaches to synergistically integrate different land uses.^25^^,^^26^^,^^27^

Wildfire-resilient landscape outcomes can be triggered by combinations of direct and indirect measures.^3^^,^^28^ Direct measures implement strategic fuel management actions (e.g., prescribed burning) to support firefighting, protect the wildland-urban interface, and increase ecosystem resilience.^22^ However, fuel management for fire risk mitigation remains scarce in many parts of the Mediterranean,^29^ and it rarely reaches the critical mass of treated area to modify landscape flammability, the fire regime, and its impacts.^21^^,^^25^ Since fuel treatment at landscape scale is untenable, the use of fire modeling and exposure assessment to optimize the location of these actions is increasingly used to reduce community risk while preserving key ecosystem services.^30^^,^^31^ Indirect measures to modify fire behavior comprise land management practices, such as sustainable forest management^32^ or agro-pastoral activities,^33^^,^^34^ that have as side effects reduced fire-hazards and improved landscape-level fire regulation capacity, by influencing vegetation type, structure, and landscape patchiness that results from the interaction between fire and biophysical structures.^22^ A strategic spatial distribution of agroforestry and agricultural land use patches can reduce overall landscape biomass content and continuity and, hence, fire intensity. It can create opportunities for safe fire extinction and, in some cases, eventually reduce suppression costs when these interventions are strategically located and designed.^23^^,^^35^^,^^36^ Furthermore, social-ecological context and place-based approaches play an important role.^5^^,^^37^ The human dimension is especially relevant since top-down fire suppression led in many places to the detachment of local people from wildfires,^38^ and in consequence local communities may be unprepared for extreme wildfire events.^14^

In this context, landscape approaches may offer a promising framework for characterizing and operationalizing wildfire risk mitigation initiatives. Landscape approaches became prominent from 1992 onwards^39^ as a response to the limitations of sectoral and fragmented management, promoting holistic, integrated, and sustainable solutions to complex, interconnected environmental and social challenges.^40^^,^^41^ Defined as governance strategies that reconcile multiple and often competing land-use objectives,^42^ landscape approaches emphasize multifunctionality, navigation of synergies and trade-offs, cross-sectoral integration, stakeholder participation, and adaptive management. Despite their growing relevance in natural resource management, the application of landscape approaches to wildfire mitigation has so far remained very limited.

Here, we argue that preventing and mitigating the increasing threats from wildfires requires a paradigm shift toward landscape approaches that can contribute to navigating uncertainties and harmonizing competing land uses through adaptive and integrated management systems.^2^^,^^4^ We aim to propose the key design features of landscape approaches to preventing and mitigating wildfires. We do so by identifying, describing, and analyzing ten wildfire mitigation initiatives in the Northern Mediterranean region as empirical examples of integrated landscape approaches. Using a structured assessment framework based on documentary evidence and expert knowledge, we assess the initiatives across key dimensions of landscape approaches. Through qualitative comparison, we identify enabling conditions, common gaps, and the barriers and facilitating factors that would allow the initiatives to replicate or expand in regions where climate and land-use changes lead to extreme vulnerability to wildfires. Existing governance structures are often difficult to change because competencies are fragmented by being shared between, for example, wildfire management, forestry, agriculture, and civil protection. However, positive incentives for governance change would include, among others, a better alignment of wildfire risk mitigation with rural development or climate-mitigation policies.^43^ The ten initiatives can be understood as local seeds^44^ with potential for transforming wildfire mitigation governance and whose broader impact depends on processes of consolidation, replication, and institutional uptake.^45^

The framework is structured around five key dimensions commonly associated with landscape approaches: multifunctionality, synergies and trade-offs, cross-sectoral processes, Participation, and Adaptive Management (Table S1). Each dimension includes specific factors, which are defined and assessed on a 4-point ordinal scale (from 1 = minimal to 4 = fully developed). This scale allows for systematic and comparative evaluation of how closely initiatives align with landscape principles. The framework was adapted from existing landscape assessment tools^46^^,^^47^ and refined to capture the specific requirements of wildfire risk mitigation in Mediterranean social-ecological contexts.

## Results

### Seeds of integrated landscape approaches to mitigate wildfires

**Figure 1:**
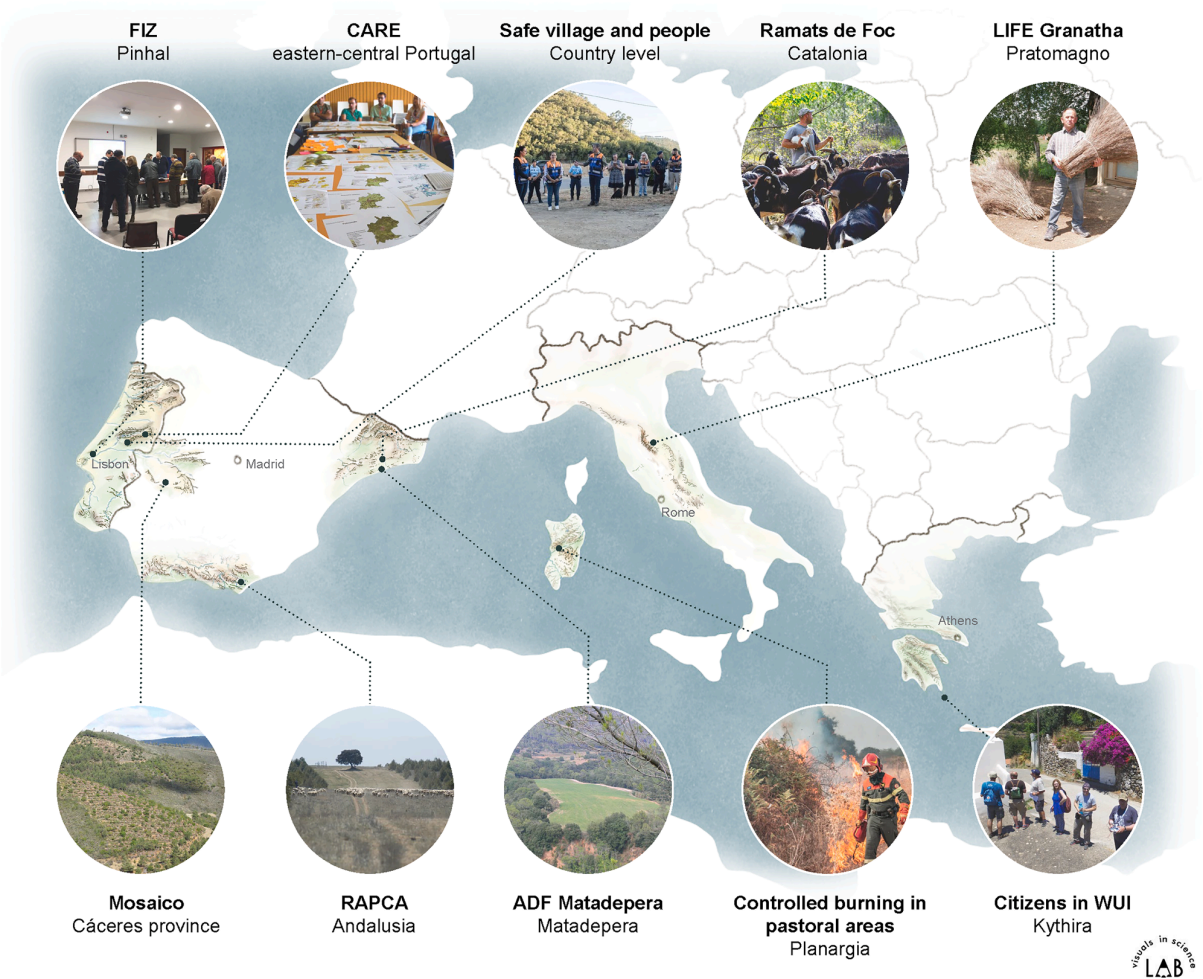
Location of the 10 selected initiatives of landscape approaches for wildfire risk mitigation

**Figure 2:**
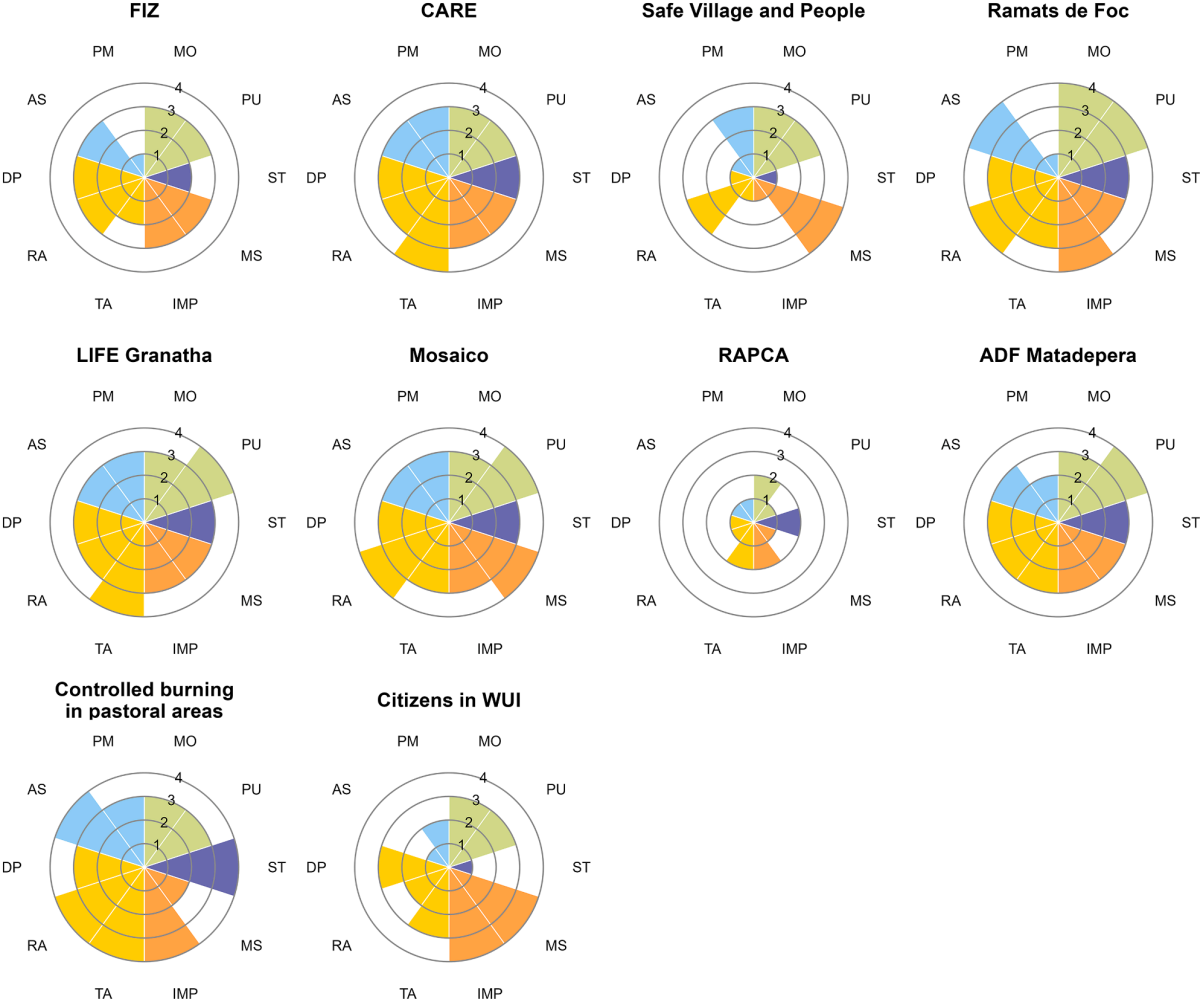
Assessment of the 10 initiatives according to dimensions and factors of landscape approach The rose diagrams display the scores of each initiative across ten key factors, grouped under five dimensions of landscape approaches. Each color represents a specific dimension:  = multifunctionality,  = synergies and Trade-offs,  = cross-sectoral,  = participation,  = adaptive management. Factor acronyms *are:* MO = multiple objectives; PU = practices undertaken; ST = synergies and trade-offs; MS = multiple-sector integration; IMP = integrated management planning; TA = transdisciplinary approach; RA = role of actors; DP = degree of participation; AS = adaptive strategy; PM = performance measurement. Scores range from 1 = minimal alignment to 4 = full achievement of the landscape approach principle.

We identified 10 local-regional level initiatives for wildfire mitigation across the Northern Mediterranean Basin (see STAR Methods). Figure 1, Box 1 and Table 1 showcase these initiatives. Although they vary in background, aims, composition, scope, and functioning, they all share the basic principles of landscape approaches. We analyzed their performance across five dimensions (operationalized by ten factors) of landscape approaches (Figure 2) to uncover critical lessons and identify pathways for scaling and transformation. A summarized description of the initiatives is provided in Table 2 while a detailed description of the 10 initiatives can be found.

Box 1 **Description of the 10 seed initiatives showcasing landscape approaches**

[1] **FIZ** (Forest Intervention Zones) is located in Pinhal Interior, one of the most fire-prone regions in Portugal. FIZs’ initial objective is promoting active forest management at the landscape level through multi-ownership collaboration in a context of small-scale ownership dominance. It was recently upgraded by the approval of long-term funding for an integrated landscape transformation in several of those FIZs.
[2] **CARE** (Collaborative Approach for Resilient Ecosystems) is an initiative in Eastern-central Portugal to promote multi-actor discussions on synergies and trade-offs of agrosilvopastoral mosaic ecosystem services in wildfire-resilient territories. It aims to support decision-making processes in the new Integrated Landscape Management Areas (AIGP) and assess the effectiveness of fire-risk mitigation actions implemented by local stakeholders by analyzing their impact on fire-regulating services.
[3] **Safe Village and Safe People** are two voluntary programs running at the national scale in Portugal, created by the Council of Ministers in 2017 and joined by the municipalities that wish to do so. The Safe Village program establishes structural measures for the protection of people and property and buildings in the urban-forest interface, with the implementation and management of protection zones for settlements and strategic infrastructures, identifying critical points and places of refuge. The Safe People program promotes awareness-raising actions to prevent risky behavior, self-protection measures, and evacuation plan drills, in conjunction with local authorities.
[4] **Ramats de Foc** (FireFlocks) is located in Catalonia, North-Eastern Spain. It promotes targeted grazing to reduce the risk of large wildfires in strategic fire management areas. The FireFlocks label was created for use in restaurants and butchers to raise awareness about wildfire risk reduction and the need to consume local products to support the shepherds and reverse rural abandonment.
[5] **LIFE Granatha** aims to reduce the loss of suitable habitat for target birds in the Pratomagno area, Tuscany Region, Italy. Conservation interventions include mechanical cutting and prescribed burning and creation of a value chain to produce and commercialize organic/biological brooms made of heathland species.
[6] **Mosaico** is a large-scale, long-term project in Extremadura, western Spain, aimed at achieving fuel load reduction through participatory landscape change by engaging all types of land managers from the agricultural, livestock, and forestry sectors.
[7] **RAPCA** is the Andalusian network of fuelbreaks maintained by targeted grazing. Its main objective is to involve local shepherds for wildfire risk mitigation in fuel breaks located in the public forests of the region. Shepherds are rewarded through a payment scheme for the service provided.
[8] **ADF Matadepera** is a municipal forest defense association in metropolitan Barcelona, Spain, working on a land management scheme aimed at building resilience to large forest fires. Actions developed include the creation of strategic areas to facilitate suppression and limit wildfire size. These areas are managed through the maintenance/recovery of cropland, thinning/pruning of forests, and extensive grazing. In addition, public outreach on wildfire risk, local landscape values, and rural heritage is conducted.
[9] **Controlled burning in pastoral areas** is located in the Central-Western area of the island of Sardinia, Italy. Started in 2012, the initiative supports local agro-pastoral farmers in land management by removing low-palatable vegetation and renewing pastures using prescribed fire in stony and marginal pastoral zones, reducing the cost of pasture management and the occurrence of arson and possibly large fires.
[10] **Citizens in WUI** (Wildland Urban Interface) are located on the island of Kythira, south of Peloponnese, Greece. The project engaged the local population to help assess the risk of destruction of individual homes in selected settlements in high-risk wildfire areas.

**Table 1:** Assessment framework for evaluating wildfire mitigation initiatives through a landscape approach

Dimensions of landscape approaches	Factors	Definitions	Rating of factors achievement			
			1	2	3	4
Multifunctionality	multiple objectives	the initiative targets an integrated set of objectives	there is only one objective	there is more than one objective but these are not integrated	there are numerous objectives integrated	there are numerous objectives integrated and continuously adapted
	practices undertaken	the initiative undertakes multiple practices to achieve its objectives (e.g., forest clearing, prescribed burning, improved labeling …)	there is only one type of practice	there is more than one practice, but focused on a single land use/actor	several practices tackling different land uses/actors	several practices tackling different land uses and dimensions beyond land uses/actors (e.g., forestry mng and value chain improvement)
Synergies and trade-Offs	consideration of synergies and trade-offs	the initiative considers synergies and trade-offs among objectives, actions and impacts	there is no consideration of either synergies or trade-offs	synergies and/or trade-offs are considered but not acted upon	synergies and/or trade-offs are considered and acted upon	synergies and/or trade-offs are considered, acted upon and periodically updated
Cross-Sectoral	multiple sector integration	different sectors related to wildfires are involved (e.g.,: forestry, agriculture, water, economy, policy)	only the forestry-related sector is involved	only the land related sectors are involved (forestry, agriculture)	other sectors beyond land-related sectors are informed but do not actively collaborate	other sectors beyond land-related sectors are informed and actively collaborate
	integrated management planning	the initiative counts with an integrated management plan	there is no plan	there is a plan but is not integrated across sectors or administrative bodies	there is a plan partially integrated across sectors or administrative bodies	there is a plan fully integrated across sectors and administrative bodies
Participation	transdisciplinary approach	the initiative includes diverse actors from the science, policy (public administration) and practice (land managers) related to wildfires	only one area of knowledge (e.g., policy)	two areas of knowledge (e.g.,. policy and practice)	three areas of knowledge	all areas of knowledge are integrated
	role	the initiative includes actors with diverse roles (power/interest) on wildfire management	only actors with power	only actors with interest	actors with power and interest	actors with power, interest, and external actors to the initiative
	degree of participation	the initiative engages multiple actors in its different phases	only informed of the initiative	informed and consulted about the initiative	actors informed, consulted and participating in the initiative development	there is an integrative strategy of participation for each phase of the initiative
Adaptive Management	adaptive	the initiative counts with a strategy to adapt periodically to changing circumstances	the initiative has not followed an evaluation and has not changed	the initiative followed a mid-term evaluation but has not been adapted	the initiative followed/is foreseen to follow a mid-term evaluation and has been adapted	the initiative follows periodic evaluations and is adapted afterward
	performance	the initiative evaluates its performance considering different social-ecological indicators	no performance measure is considered	performance is based on a single metric	performance is measured considering several metrics	performance is measured considering several metrics that encompass short- and long-term performance and/or have been agreed with stakeholders

**Figure 3:**
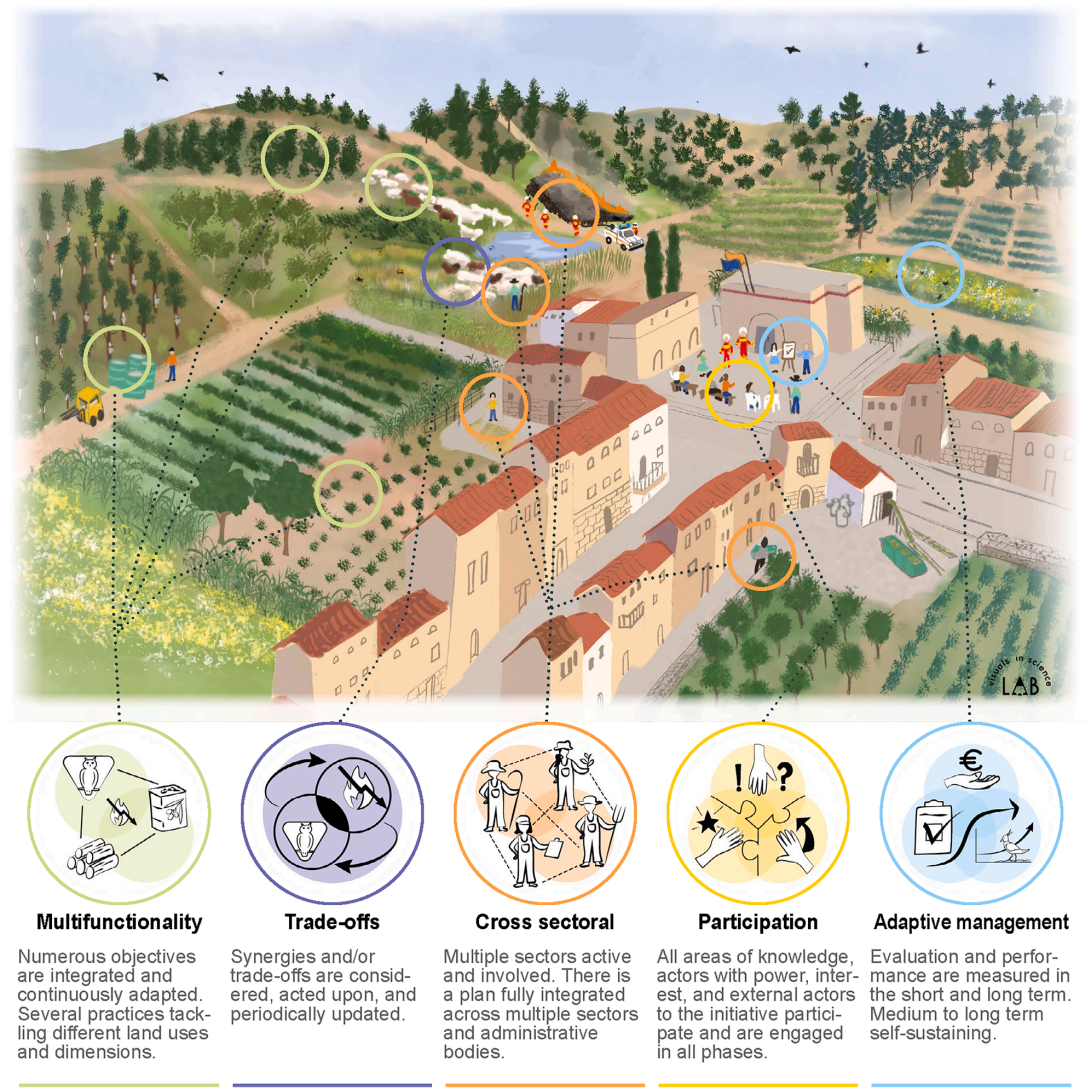
The manifestation of core landscape approach dimensions in fire-prone Mediterranean landscapes

**Table 2:** Characterization of the 10 seed initiatives showcasing landscape approaches

Initative	Geographic extent	Administrative/territorial scale	Land tenure and context	Main objective and practices	Funding model	Partners and agencies involved	Status/timeline
**[1] FIZ** (Forest Intervention Zones)	774 km^2^ (26 FIZ)	regional/municipal	non-industrial private forests dominant; landscape homogenization (forest/scrubland 83%).	active forest management; fuel breaks, partial maintenance, integrated landscape transformation	shifted from initial 2-year establishment to yearly funding	Forest Authority, General Directorate for Territory, Forest Owners Associations	active (since 2006–2011/2021)
**[2] CARE** (Collaborative Approach for Resilient Ecosystems)	2,087 km^2^ (3 municipalities: Sabugal, Penamacor, Fundão)	regional/municipal	forest-dominated (44–60%); mosaic of mixed woodland and pasture.	assess fire-risk mitigation impact; promote multi-actor discussion; co-create nature-based solutions (NbS).	structural protection of buildings/settlements (Village); awareness/evacuation drills (people).	management plan has 20-year funding; CARE supported by competitive projects.	active
**[3] Safe village and safe people**	national scale	national scale	applicable in diverse areas with variable land use characteristics.	structural protection of buildings/settlements (village); awareness/evacuation drills (people).	Silvopastoralism; graze strategic management areas to reduce fuel; local labeling (FireFlocks).	created without a specific dedicated budget (Council of Ministers Res. 157-A/2017).	active (Since 2017)
**[4] Ramats de Foc**	Regional (Catalonia, Spain)	regional	rural abandonment; secondary vegetation proliferation.	Silvopastoralism; graze SMAs to reduce fuel; local labeling (FireFlocks).	Pau Costa Foundation; regional firefighting services, shepherds,	payments by results (subsidies) from Rural Development Program	active
**[5] LIFE Granatha**	21,050 ha (Pratomagno mountain range)	regional	abandonment of traditional activities; 27.6% private, 72.4% public lands	reduce habitat loss (birds); fuel reduction; create value chain for heathland species	LIFE program; prescribed burning; private/public funds.	DREAM Italia, local private owners, municipalities	completed (2017–2023)
**[6] Mosaico**	Sierra de Gata (111k ha) and Las Hurdes (46k ha).	regional	forestland dominant (91%/81%); high fire recurrence/abandonment	fire smart territory; participatory landscape change; direct/indirect prevention.	public/private and competitive funding	land managers (agri, livestock, forestry sectors), University of Extremadura	active
**[7] RAPCA**	Andalusia (5,433 ha grazed).	regional	public forests (regional and municipal ownership).	targeted grazing in fuelbreaks to reduce mechanical cleaning costs.	budget from wildfire prevention services (INFOCA)	regional government, local councils, 169 shepherd contracts	active
**[8] ADF Matadepera**	25.4 km^2^ (residential suburb of the Barcelona Metropolitan Region).	municipal	low-density wildland-urban interface; natural park adjacency.	build resilience; fuel load reduction; land management (cropland/thinning/grazing)	public municipality, region) and private	ADF (group for forest defense), municipal government	active
**[9] Controlled burning in pastoral areas**	Planargia area (Suni, Oristano) 4,800 ha	local/municipal	agro-pastoral; stone/rocky marginal pastoral zones	reduce uncontrolled/escaped fire; renew pastures; reduce costs	public	Sardinia Forest Service, agro-pastoral farmers	active
**[10] Citizens in WUI** (wildland urban interface)	Island of Kythira (277.28 km^2^)	municipal	high-risk wildfire areas (phrygana/evergreen shrubs)	assess risk of individual homes; population awareness	research/project funding	Hellenic Society for Protection of Nature, Institute of Mediterranean forest ecosystems of ELGO DIMITRA	ended

Check Supplementary material for more information on each initiative.

### Wildfire mitigation initiatives sprouted through a common concern entry-point

The aftermath of big wildfire events may serve as a tipping point, bringing together key stakeholders with a common concern for shifting from the business-*as*-usual focus on fire suppression^48^ toward actions that promote more resilient landscapes to wildfires. Such a common-concern entry point underpinned the emergence of several initiatives such as Mosaico in western Spain [6], FIZ in Portugal [1] or controlled burning in pastoral areas in Sardinia (Italy) [9]. Mosaico [6] strove to recover active traditional land uses beyond inertia-driven postfire pine afforestation. Several disastrous wildfire seasons that reverted forest transition in Portugal^49^ prompted the conception of FIZ [1], an innovative modality of multi-ownership collaboration to tackle forest land abandonment in a context of small ownership dominance, aiming to overcome land ownership fragmentation. Initiative [9] built bridges between the Forest Service and traditional agro-pastoralists to conduct controlled burning in the areas demanded by the latter, based on the shared acknowledgment of the usefulness of fire as a tool to renew pastures while lowering the occurrence of clandestine fires typically set up in total ban periods (e.g., September) where use of fire is forbidden.

### Dimension 1: multifunctionality pursued: wildfire mitigation and broader landscape objectives

Landscape approaches aim to achieve multiple objectives in an integrated manner, striving for improved synergies and minimized trade-offs and increasing overall landscape multifunctionality. Although mitigating wildfire risk is always an important (if not the most important) goal, many of the analyzed initiatives aim for multiple synergistic goals. LIFE Granatha [5] exemplifies how synergies between open habitat restoration for enhanced bird diversity conservation can show beneficial effects for wildfire risk mitigation. Mosaico [6] develops profitable agroforestry activities to create a mosaic of multiple land uses that could contribute to halting depopulation and increasing biodiversity linked to open and semi-open landscapes. The conservation of open landscapes may also entail objectives related to cultural heritage. For example, research and outreach can promote local memory of the landscape mosaics of the past, which serve as a symbolic source of inspiration for fuel-reduction activities, as shown by the initiative ADF Matadepera [8]. Striving for multiple objectives usually implies developing a wide range of practices that may span beyond traditional land use-related actors and standard biomass removal for wildfire risk mitigation. Ramats de Foc [4] aims to improve the financial viability of grazing as a tool for biomass reduction by engaging with local groceries and improving the market share and margin of shepherds’ meat while promoting payment schemes linked to the ecosystem services provided by extensive grazing. Across the sampled initiatives, multifunctionality showed as a more salient feature when fuel reduction was treated as part of a broader reconfiguration of land-use mosaics rather than as an isolated task.

### Dimension 2: synergies are more visible than trade-offs

Several initiatives showcase how synergies can be generated between wildfire mitigation and other ecological or socio-economic goals. However, we found that explicit attention to trade-offs was less common although trade-offs are likely to arise between fire-regulating services and other ecosystem services. In this sense, the CARE initiative in Portugal [2] stands out because it will undertake an analysis to identify ecosystem services synergies and trade-offs. In contrast, in many other initiatives, trade-offs appeared to be acknowledged only implicitly or remained weakly monitored.

### Dimension 3: cross-sectoral processes shape wildfire management

Several initiatives involve stakeholders from different sectors. These initiatives are guided by integrated management planning that orchestrates sectoral participation. LIFE Granatha [5] engages civil protection actors, local land management agencies with competences on wildfire management and volunteer fire brigades as well as key economic actors to develop the local supply chain of organic heath brooms. An integrated wildfire risk mitigation plan clarifies the roles of the different actors in the different actions, from habitat conservation to value chain creation. In Ramats de Foc [4], shepherds are involved together with the more traditional forestry and emergency-related actors. Similarly, these actors are guided by a strategic document that clearly outlines roles and responsibilities of each of them. In the FIZ initiative [1], planning documents are being modified to involve sectors traditionally not engaged in wildfire protection, such as energy and tourism, beyond the local forestry landowners.

### Dimension 4: meaningful participation as core element

Most initiatives present a high degree of participation that implies the information, consultation and active involvement of a range of actors in the development of the initiative. In the CARE initiative [2], participating actors are engaged to discuss potential synergies and trade-offs between the fire-regulating ecosystem services and other key ecosystem services. Additionally, several areas of knowledge are combined in a range of initiatives, including scientific, policy, and practice stakeholders. Researchers participating in LIFE Granatha [5] undertake monitoring of different landscape management actions according to agreed indicators. In Mosaico [6], researchers assess initiative performance in terms of improved resilience to wildfires, while in Controlled burning in pastoral areas [9], they assess metrics after prescribed burnings in terms of reduction of burnt area, number of wildfires, and suppression costs (e.g., hours of aerial interventions for firefighting). New boundary-spanning organizations may emerge that allow leading community members to network with key actors while leveraging their expertise and local knowledge, as shown in ADF Matadepera [8]. Participation and transdisciplinary integration therefore appear to be central features of landscape coherence.

### Dimension 5: adaptive management remains uneven and linked to scientific involvement

Adaptive management approaches are found in some of the selected initiatives. In RAPCA in Spain [7], adaptive management was endorsed in the early set up phases to develop the result-based payment scheme for targeted grazing linked to a monitoring procedure to verify performance levels in biomass reduction by livestock. However, the perceived success in the short to medium term, combined with institutional inertia, has prevented the adoption of a mindset focused on learning and adaptation.^50^ In contrast, Ramats de Foc [4] has shifted from an initial focus on livestock product valuation to exploring new strategies, namely political lobbying to promote payments for ecosystem services as a result of continuous evaluation of initiative performance with shepherds.^51^ Adopting a performance protocol is sometimes eased by the involvement of research organizations in the initiatives. how to attribute reduced number or intensity of wildfires to prescribed burning conducted). Researchers’ involvement can challenge the status quo maladaptive practices such as exemplified by the Citizens in WUI in Greece [10] where researchers fostered fire risk analysis as part of regular practices by public administrations. In ADF Matadepera [8] continuous observation of the performance of strategic management areas in terms of fuel structure is undertaken to identify additional necessary interventions. In FIZ [1] the “informal” assessment of the (low) effectiveness of interventions strictly focused on reducing biomass in forest land is leading to the adoption of integrated landscape transformation measures (i.e., setting thresholds of forest cover at landscape scale and promoting open farmland areas).

### Scoring reveals uneven but informative patterns

Our analysis of the ten initiatives based on the proposed landscape approach framework reveals notable patterns (Figure 2). First, we observe that some initiatives perform well in all dimensions of the landscape approach. Initiatives such as Mosaico [6], Ramats de Foc [4], ADF Matadepera [8], and CARE [2] score consistently well across most or all dimensions, integrating multiple objectives and practices, engaging diverse stakeholders, and demonstrating strong transdisciplinary approaches. In contrast, FIZ [1] and Safe Village and People [3] show more uneven accomplishment. While they perform relatively well in multifunctionality and participation, they fall short in addressing synergies and trade-offs and adaptive management, highlighting potential challenges in cross-sectoral coordination and strategic alignment too. RAPCA [7] and Citizens in WUI [10] stand out for their more limited scope. Their low scores in adaptive management, cross-sectoral integration, and multifunctionality suggest that these initiatives are either in early development stages or are more narrowly focused. RAPCA [7], in particular, shows some ineffectiveness, lacking strength across nearly all dimensions, while Citizens in WUI [10] shows participatory depth but limited institutional integration and strategic planning. The factors of degree of participation (including the diversity of actors and depth of engagement) and transdisciplinary approach seem to be heterogeneous among initiatives. Strong performers consistently demonstrate meaningful involvement of multiple actor types and knowledge systems, which aligns with greater landscape coherence. Across many initiatives, synergies and trade-offs, and attainment in the adaptive management dimension, tend to score low. This reflects a broader challenge linked to the need for better tools and processes to evaluate outcomes and mediate between competing land-use objectives. Even advanced cases often lack systematic indicators or mechanisms to assess effectiveness across ecological and social goals.

## Discussion

### Landscape approaches as a possible pathway to reframe wildfire governance

The ten seed initiatives analyzed in this study highlight the importance of landscape approaches in addressing wildfire risk while simultaneously advancing multiple sustainability goals. By fostering synergies between conservation, sustainable land use, and community engagement, these projects aim to go beyond conventional fire suppression strategies.^52^ They offer a promising pathway toward landscapes that are not only more fire-resilient but also more biodiverse, economically viable, and culturally meaningful.

Landscape approaches orchestrate cross-sectoral participation and collaboration from many different sectors, involving a wide set of actors that bring in different types of knowledge and transdisciplinary processes (Figure 3). Such actor diversity supports mutual learning among scientists and practitioners and blending of local, technical, and scientific knowledge.^53^ In the case of wildfires, the high interdependency between forest management, civil protection policies, land-use planning, agriculture development, climate, and energy policies impacting fire management calls for a move away from the currently narrow focus on fuel management within the forestry sector only.^14^^,^^54^ The analyzed initiatives exemplify the approach advocated by many scholars^36^^,^^54^^,^^55^ for cross-sectoral participation and inclusion, which requires the development of planning tools to delineate the roles of the different actors in an effort to involve sectors and actors traditionally left out of the wildfire problem. This is relevant because, whereas the aims of reducing risk and increasing fire-resilience in the landscape may remain the same, the pathways to achieving them can differ across contexts.^15^^,^^56^^,^^57^

Our analysis provides some ideas of how to bridge community-level approaches with professional wildfire fighting services, which may ultimately improve trust between these actors.^58^ For instance, RAPCA [7] or Ramats de Foc [4] showcase the collaboration between firefighting services in delimitation of strategic areas for wildfire suppression and shepherds, where targeted grazing is employed to maintain low biomass content. ADF Matadepera [8] exemplifies how local volunteer groups can be engaged in wildfire risk mitigation, in ways that improve their relationship with wildfire management actors. Finally, LIFE Granatha [5] and Controlled burning in pastoral areas [9] illustrate how prescribed burning undertaken by professional firefighting services can restore mosaic landscapes for enabling the recovery of traditional activities.

### Adaptive management and the role of science

Landscape approaches are long-term engagements, but short-term process metrics are needed to confirm that progress is being made in terms of negotiation of goals, meaningful stakeholder engagement, existence of connections to policy processes, and effectiveness of governance.^59^^,^^60^ Evaluation is thus a fundamental feature of adaptive management. The results of fire management actions are to be assessed and monitored regularly, aiming at continuous improvement based on the identification of (and building on) the lessons learned in the process.^61^ Key performance indicators need to combine process and outcome metrics, considering governance-related measures (e.g., stakeholder participation, cross-sector coordination) and fire-related outcomes^62^ (e.g., reduced fire exposure, lower fire sensitivity, reduced losses to people and assets and improved community preparedness), so that evaluation of policy effectiveness transcends the sole consideration of burned area.^63^^,^^64^

Adaptive management is linked to landscape approaches since the latter aims at improving environmental management and generating knowledge through systematic learning from management outcomes.^65^ This ultimately may require venturing into unknown paths with a high level of uncertainty, and hence it is inextricably linked to performance measurement to enable its inherent learning-by-doing approach. Adaptive management encourages the development and testing of diverse, tentative, and learning-oriented solutions,^3^ but it remains marginal in fire-prone ecosystems because it requires a profound institutional change^66^ and sustained time and resource investments from partners. However, its implementation is a necessary process for facilitating change in the predominant fire-suppression-centered management paradigm.^60^^,^^67^^,^^68^

Several initiatives in this study (e.g., Mosaico [6], LIFE Granatha [5]) stand out for the set-up of monitoring protocols that involve scientists and managers, allowing for continuous evaluation and adaptive management. Such protocols can also enhance local capacities and establish continuous learning processes that include different viewpoints and help negotiate knowledge.^69^ However, this also highlights the challenge of sustaining stakeholder engagement beyond initial project phases. Single-contact coordination and project-based funding may jeopardize the depth and duration of stakeholder participation, underscoring the need for arrangements that provide ongoing time and resource support for collaborative learning.^70^

### Scaling landscape approaches from local initiatives to systemic change

Evidence from the ten initiatives in the Mediterranean shows that landscape approaches may act as seeds of transformation^44^ and have the potential to shift prevailing wildfire management paradigms toward better thriving with fire. However, advancing landscape approaches in wildfire policy requires greater investment in collaboration, shared learning, and long-term governance capacities. In particular, broadening their impact depends on processes of amplification^45^ within, out of, and beyond the initiatives.

First, amplifying within encompasses processes that increase the impact of a specific initiative by stabilizing its existence.^45^ This is frequently linked to securing medium- to long-term funding, which can be challenging for integrated landscape initiatives.^46^ Additionally, improved performance of the initiatives according to the dimensions and factors proposed may imply more complexity in project management linked to high levels of cross-sectoral integration and participation. This will require a commensurate effort in the total amount and longevity of resources applied to the engagement. From the selected initiatives, lessons can be extracted on how to prolong the impact of integrated landscape initiatives. This might be done, for instance, by linking them to the relatively stable earmarked regional budgets for wildfire management or by developing profitable agroforestry-based management alternatives that can be self-sustained with the revenue obtained from their products, as found in some of the initiatives with higher levels of cross-sectoral integration.

Second, processes based on amplifying out replicate and adapt initiatives across different contexts, acknowledging that the coevolution of people with wildfires can look dramatically different across cultures and landscapes,^71^ and thus reflecting the diversity of institutional legacies found in Northern Mediterranean countries.^25^ Therefore, amplifying out the initiatives typically requires addressing local risk perceptions, informal rules, social norms, and landowner opportunity costs for risk-preventive actions. An example of this tailoring is found in Ramats de Foc [4], where biomass reduction through controlled grazing was adopted from RAPCA [7] and further adapted by establishing alliances with local butchers to revalorize meat products from the involved flocks.

Third, amplifying beyond consists of processes that generally seek to increase impact by scaling up to reach higher institutional levels or by scaling deep to change societal values. Amplifying beyond ultimately refers to the extent that the initiatives can contribute to the advocated need for a paradigm shift in wildfire management. For systemic transformation to occur and endure, changes must take place both at the institutional level and in societal behavior, which is shaped by personal values and social norms.^72^^,^^73^^,^^74^ These two dimensions engage in mutual feedback: Institutions establish the legal frameworks and incentive structures that influence behavior.^75^^,^^76^ While behavioral change operationalizes institutional reforms.^77^ Several initiatives in our study illustrate this interplay. For instance, the Controlled burning in pastoral areas initiative [9] demonstrates how institutional reform—specifically, enabling a demand-driven approach to prescribed burning—altered farmer behavior and significantly reduced arson-related wildfires. Amplifying beyond in terms of institutionalization occurs when such initiatives are integrated into public administration and policy frameworks. This process often requires new legislation or intergovernmental coordination and changes across multiple levels—regulative (rules and laws), normative (social values), and cognitive (shared beliefs)^78^—where results do not merely stem from rational planning but from negotiations, contestation, and political compromise.^79^ Tensions are especially salient in adaptation efforts, such as those involving wildfire management, which often bring competing priorities of public and private actors into conflict.^80^

While a full assessment of institutional adaptability is beyond the scope of this study, initiatives like ADF Matadepera [8], controlled burning in pastoral areas [9], and FIZ [1] may offer valuable insights into how such conflicts can be navigated to enable institutional transformation. Our findings highlight the need for future research on how institutional frameworks can enhance societal adaptive capacity^81^ and how environmental changes drive institutional innovation.

### Limitations of the study

Several limitations should be acknowledged in this study. First, the purposive sampling of ten initiatives limits the ability to generalize our findings beyond the cases analyzed. The initiatives act as illustrative examples rather than being representative of all wildfire mitigation efforts in one region. Nevertheless, our assessment identified patterns across initiatives and provided insights on how similar local-regional projects or processes might adopt landscape approach logics. Second, the assessment is based on interpretative expert scoring. Although efforts were made to ensure consistency and transparency throughout the process through a structured and detailed assessment framework as well as joint validation, as a qualitative methodology it still involves the subjectivity of individual expert judgment. Third, the assessment framework focuses on the processual and design features of the initiatives and does not allow for evaluating the performance of the initiatives under wildfire conditions. Such evaluation would require quantitative impact assessments involving longitudinal data (e.g., see Pulido et al. 2023^36^). While this is beyond the scope of this study, examining the relationship between landscape-approach dimensions and observed wildfire outcomes would be a valuable future research avenue to improve effectiveness and efficiency of these initiatives for wildfire risk mitigation.

## Resource availability

### Lead contact

Requests for further information and resources should be directed to and will be fulfilled by the lead contact, Elsa Varela (elsa.varela@csic.es).

### Materials availability

This study did not generate new materials.

### Data and code availability

• All data reported in this article are found in the main manuscript and the supplementary material and are publicly available as of the date of publication.
• This paper does not report original code.
• Any additional information required to reanalyze the data reported in this paper is available from the lead contact upon request.

## Acknowledgments

This research has been funded by the 10.13039/501100001659Deutsche Forschungsgemeinschaft (DFG, 10.13039/501100001659German Research Foundation), project number 426675955. Elsa Varela is grateful to Pastores por el Monte Mediterráneo Association for updating the information on the RAPCA initiative. Elsa Varela acknowledges the support of the 10.13039/100005156Humboldt Foundation. Iago Otero is grateful to Ricard Farriol for providing information on the ADF Matadepera initiative. Davide Ascoli contributed to this study within the Agritech National Research Center and received funding from the European Union Next-Generation EU (PIANO 763 NAZIONALE DI RIPRESA E RESILIENZA (PNRR) – MISSIONE 4 COMPONENTE 2, INVESTIMENTO 764 1.4 – D.D. 1032 17/06/2022, CN00000022. Fernando Pulido acknowledges funding from the 10.13039/100018693European Union’s Horizon Europe program under grant no. 101093968 (10.13039/100015034Resist). Michele Salis and Valentina Bacciu contributed to this study within the projects MED-Star2 (grant no. B83C24005570007), funded by the EU under the cross-border “Programma Italia–Francia Marittimo” 2021–2027, and FIRE-ADAPT (grant no. 101086416), funded by the EU under the program MSCA Staff Exchanges.

## Author contributions

Conceptualization: E.V. and T.P.; methodology: E.V., M.H.-M., and M.J.; investigation and formal analysis: E.V., D.A., M.A., V.B., M.J.C., G.C., P.C., I.O., A.P., F.P., N.P., M.S., F.T., and G.X.; data curation: E.V.; writing – original draft: E.V.; writing – review: E.V., M.J., T.P., M.H.-M., D.A., M.A., V.B., M.J.C., G.C., P.C., I.O., A.P., F.P., N.P., M.S., F.T., and G.X.; funding acquisition, T.P.

## Declaration of interests

The authors declare that they have no competing interests.

## Declaration of generative AI and AI-assisted technologies in the writing process

During the preparation of this work, the author(s) used ChatGPT in order to improve English language grammar and readability. After using this tool/service, the author(s) reviewed and edited the content as needed and take(s) full responsibility for the content of the publication.

## STAR★Methods

### Key resources table

**Table:** 

REAGENT or RESOURCE	SOURCE	IDENTIFIER
**Deposited data**		

Scoring data for the 10 initiatives	This paper	N/A

**Software and algorithms**		

R statistical software	R Core Team	RRID: SCR_001905; https://www.r-project.org/
ggplot2 R package	Wickham et al.^82^	https://CRAN.R-project.org/package=ggplot2
tidyr R package	Wickham et al.^83^	https://CRAN.R-project.org/package=tidyr
dplyr R package	Wickham et al.^84^	https://CRAN.R-project.org/package=dplyr
scales R package	Wickham et al.^85^	https://CRAN.R-project.org/package=scales
geomtextpath R package	Cameron and van den Brand^86^	https://CRAN.R-project.org/package=geomtextpath
cowplot R package	Wilke^87^	https://CRAN.R-project.org/package=cowplot
ChatGPT	OpenAI	https://openai.com/chatgpt

### Method details

#### Study design

This study used a comparative qualitative research design to assess whether and how existing wildfire mitigation initiatives in the Northern Mediterranean region reflect key dimensions of landscape approaches. The analysis focused on ten initiatives operating at local to regional scales in Portugal, Spain, Italy, and Greece.

The study was not designed to test the effectiveness of each initiative during wildfire events (i.e., reduction of wildfire severity or damages), but rather to evaluate the extent to which these initiatives incorporate relevant features associated with landscape approaches and that may support more coherent, adaptive, and socially embedded wildfire risk mitigation.

#### Conceptual and assessment framework

The need for integrated social-ecological approaches to wildfire mitigation resonates well with the notion of “landscape approaches”, defined as “governance strategies that attempt to reconcile multiple and conflicting land-use claims to harmonize the needs of people and the environment and establish more sustainable and equitable multi-functional landscapes”.^42^ Across the world, landscape approaches have assumed a crucial role in triggering a paradigm change of integrative natural resource management and enabling landscape-scale strategies.^88^ Several global and continental reviews identified more than 1000 integrated landscape initiatives^46^^,^^89^ and described them as hubs of collaborative and place-based landscape management. However, landscape approaches have been rarely used for landscape-level wildfire management so far However, wildfire management has not been a prominent aim among these landscape initiatives so far.^46^

Over the decades, the growing recognition of the interdependence between biodiversity conservation, agricultural production, ecosystem services, and human well-being led to the understanding of entire landscapes as dynamic, interrelated systems. Various principles of landscape approaches have been proposed.^47^^,^^59^^,^^90^ In this article, we focus on the following key features of landscape approaches: (a) Common-concern entry points; (b) Multifunctionality, synergies and trade-offs; (c) Stakeholder negotiation; and (d) Learning, flexibility, and adaptation. These four principles were selected because they capture core dimensions that are particularly relevant for wildfire risk mitigation in complex and dynamic socio-ecological systems. First, the feature of common-concern entry points reflects the need to align diverse stakeholder interests around shared landscape challenges, such as increasing wildfire risk. Second, the feature of multifunctionality, synergies, and trade-offs acknowledges the importance of balancing fire prevention with other land-use objectives (e.g., biodiversity conservation, grazing, or rural livelihoods). Third, stakeholder negotiation is essential in contexts where competing claims and governance asymmetries require inclusive dialogue and joint decision-making. Finally, learning, flexibility, and adaptation are critical for enabling responsive management in the face of ecological uncertainty, changing climate conditions, and evolving social dynamics. Together, these features provide an integrated framework to assess how current wildfire mitigation initiatives align with the tenets of landscape approaches. We operationalized this conceptual framework using an assessment framework that combines five landscape approach dimensions (Figure 1 and Table S1): multifunctionality, synergies and trade-offs, cross-sectoral processes, participation and adaptive management.

The dimension of multifunctionality refers to the achievement of multiple objectives or functions at the same time^91^ and this is also linked to undertaking multiple management practices to achieve several objectives. Landscape approaches are multifunctional by acknowledging the dynamic nature of landscapes and harnessing synergies. However, instead of assuming win-win outcomes, landscape approaches frame realistic objectives while recognizing and navigating trade-offs to be able to achieve multifunctionality within landscapes to increase benefits and mitigate conflicts among all stakeholders.^47^ Synergies and trade-offs therefore represent the second dimension in our assessment framework. Third, landscape approaches are driven by cross-sectoral processes. Adopting a multifunctional approach to management implies working across sectors to ensure that knowledge and information as well as land uses, markets, and policy strategies are adequately integrated.^39^^,^^91^ One of the rationales for taking a landscape approach is its ability to move beyond traditional sectoral approaches. These have often failed to adequately consider the interests and needs of other sectors. As a result, they fall short in addressing complex problems that require ongoing decision-making. Such problems are typically characterised by high levels of uncertainty, the absence of clear-cut solutions, and interactions across multiple spatial and temporal scales. They also involve a diverse set of stakeholders with differing values and priorities.^91^ This cross-sectoral integration requires integrated management planning for orchestrating the actions of the different sectors, their roles and responsibilities. Landscape approaches emphasize transdisciplinary approaches and stakeholder negotiation related to resource management and/or environmental goals,^91^ and participation build the fourth dimension of our assessment framework. Finally, landscape approaches recognize the importance of adaptive management processes implemented through regular and ongoing negotiation, which allows for continuous reflection.^59^ Adaptive management is a systematic approach that aims at improving environmental management and building knowledge through systematic learning from management outcomes.^92^ Performance evaluation of management actions is required so that adjustments are made on actions based on the performance results. During the adaptive management process, in contrast to traditional management, change is welcomed, learning is emphasized, and even goals and objectives for the project may be revisited and revised.^93^ Adaptive management feedback mechanisms provide stakeholders the capacity to best account for conservation and development challenges within the landscape.

In order to explore place-based wildfire mitigation initiatives, we further operationalized the five dimensions of landscape approaches by defining specific factors and their levels of achievement (Figure 1 and Table S1).

#### Case identification and selection

The coordinating team identified ten initiatives that address wildfire mitigation in the Northern Mediterranean Basin through either their own first-hand knowledge or knowledge through scientific publications. Initiative selection was purposive rather than statistically representative since the intention was to identify initiatives that went beyond narrowly technical or sectoral fire management.

Eligible initiatives had to meet three conditions: (1) wildfire mitigation or prevention had to be an explicit objective, either primary or strongly connected to other land-management goals; (2) the initiative had to involve interaction among different actors, land uses, or sectors at a landscape or territorial scale; and (3) an expert willing to co-author the manuscript and with first-hand knowledge had to be available to ensure sufficient documentary and expert information were available for the systematic assessment.

#### Data sources

Each initiative was assessed using documentary evidence and expert knowledge. Documentary sources included project materials, institutional and policy documents, websites, technical reports, and other written sources available to the research team. These materials were complemented by the expert knowledge of co-authors and collaborators familiar with the selected initiatives. The combination of documentary evidence and case-specific expertise was necessary because many of the dimensions under analysis—such as degree of participation, integration across sectors, or adaptive learning—are not fully observable through public project descriptions alone. The goal was not to generate a complete ethnographic account of each initiative, but to assemble sufficient evidence to support a structured comparative assessment.

#### Operationalization of the assessment framework and scoring process

The five dimensions were translated into specific factors. Each initiative was assessed on a 4-point ordinal scale for each factor, where 1 indicates that the factor is not present or minimally recognized; 2 indicates particle recognition or unfolding of the factor; 3 indicates substantive implementation with the factor clearly embedded in the initiative; and 4 indicates full achievement of the landscape approach principle. The dimensions and factors are summarised in Figure 1. The framework assessed whether initiatives:

• pursued multiple objectives and used diverse practices (multifunctionality),
• explicitly recognized or addressed synergies and trade-offs among landscape objectives,
• integrated sectors and management domains (cross-sectoral processes),
• involved diverse actors and meaningful forms of engagement (participation), and
• incorporated monitoring or feedback mechanisms (adaptive management).

The ordinal scale employed intended to serve as an interpretative and comparative tool to assess to what extent the initiatives operationalized key dimensions of landscape approaches. Inferring causality as to the effectiveness of the initiatives in the event of a wildfire is out of the scope of this work, where focus is descriptive and comparative rather than predictive. Furthermore, given the purposive case selection, the small number of initiatives, and the interpretive character of several dimensions, the analysis does nsupport statistical generalization.

Each initiative was represented by at least one of the paper co-authors. First, they characterized individually their own initiative through a qualitative description of the performance of the initiative for each of the five dimensions and respective factors above described (Figure 1). Then, they scored the initiative according to the assessment framework provided and the rating of factors achievements provided (Figure 1). This first round of scoring process was conducted independently by each initiative representative. Descriptive analysis and scoring for each initiative was reviewed by the coordinating team for consistency. Identified inconsistencies were then jointly reviewed and validated through discussion among the authors and with the coordinators of the study to ensure consistency and shared interpretation. The final scores reported in Figure 3 represent the outcome of this joint validation process.

### Quantification and statistical analysis

This study employed a structured qualitative scoring approach to asses each of the ten wildfire mitigation initiatives against ten factors grouped under five dimensions of landscape approaches, using a 4-point ordinal scale (1 = minimal alignment; 4 = full achievement). Scores were assigned independently by the co-author(s) with first-hand knowledge of each initiative and subsequently reviewed and validated through discussion with the coordinating team to ensure consistency of interpretation across cases.

Given the purposive case selection, the small number of initiatives (*n* = 10), and the interpretive character of several dimensions, the scoring data do not support inferential statistical generalization. The scores are used descriptively and comparatively to identify patterns across initiatives. No measures of central tendency, dispersion, or statistical significance testing were applied.

Rose diagrams displaying the scores of each initiative across the ten factors (Figure 2) were produced using R (version 4.5.1 “Great Square Root”) with the packages ggplot2, tidyr, dplyr, scales, geomtextpath, and cowplot. All scores used to produce Figure 2 are reported.

## Supplemental information

Document S1. Table S1 and supplementary material

Data S1. ILA_Adapted_Wildfires_19062026
