# Supplementary material for: Capitalizing on integrated landscape approaches for wildfire risk mitigation in the Mediterranean region
Source: iScience. 2026 Jul 28;29(8):116741. doi: 10.1016/j.isci.2026.116741 (PMC13450235; doi:10.1016/j.isci.2026.116741)
Supplement: Supplementary file 1 [file mmc1.pdf]

iScience, Volume *29*

Supplemental information

Capitalizing on integrated landscape approaches for wildfire risk mitigation in the Mediterranean region

Elsa Varela, Mónica Hernández-Morcillo, Marion Jay, Davide Ascoli, Miltiadis Athanasiou, Valentina Bacciu, Maria João Canadas, Guillem Canaleta, Paula Castro, Iago Otero, Anabela Paula, Fernando Pulido, Núria Prat, Michele Salis, Fantina Tedim, Gavriil Xanthopoulos, and Tobias Plieninger

Table S1. Assessment framework of landscape approach dimensions and factors. Bibliographic quotes supporting the selected dimensions and factors

**Landscape approach dimensions**	**Landscape approach factors**	**Bibliography supporting the selected dimensions and factors**
**MULTIFUNCTIONALITY**	Multiple objectives	• Framed around the concept of multifunctionality, with the aim of achieving multiple objectives through the approach[S1, S2].<br>• Multifunctionality: achieving multiple objectives or functions at the same time[S2]<br>• Shared or agreed-upon management objectives that encompass multiple landscape benefits[S3]
	Practices undertaken	• Field practices that are designed to contribute to multiple objectives[S3]
**SYNERGIES & TRADE-OFFS**	Consideration of synergies and trade-offs	• Ecological, social and economic dimensions explicitly acknowledged, including trade-offs across dimensions[S1, S2, S4]<br>• Management of ecological, social, and economic interactions for the realization of positive synergies, and the mitigation of negative trade-offs[S3]
**CROSS-SECTORAL**	Multiple sector integration	• Sectoral approaches considering activities of other sectors; cross-sectoral approaches addressing cross-sectoral objectives[S2]
	Integrated management planning	• This "integrated" approach to realizing sustainable landscapes is a fundamental precept of sustainable landscape management[S5]
**PARTICIPATION**	Transdisciplinary approach	• Transdisciplinarity: collaborative integrated approaches are needed to address complex landscapes[S2]
	Role	• Type of participation: stakeholders involved in planning phases, in the development of the action, in its impact evaluation[S1, S2, S4]
	Degree of participation	• Achieve improvements by engaging and empowering the people who are affected[S6]

**ADAPTIVE MANAGEMENT**	Adaptive	• Continual learning and adaptive management[S1]<br>• Learning, flexibility and adaptation[S6]<br>• Monitoring of the landscape to understand emergent patterns and processes[S2]
	Performance	• Participatory and user-friendly monitoring[S1]<br>• Evaluate progress[S7]

REFERENCES

S1. Sayer, J., Sunderland, T., Ghazoul, J., Pfund, J.L., Sheil, D., Meijaard, E., Venter, M., Boedhihartono, A.K., Day, M., Garcia, C., et al. (2013). Ten principles for a landscape approach to reconciling agriculture, conservation, and other competing land uses. Proceedings of the National Academy of Sciences of the United States of America *110*, 8349–8356. https://doi.org/10.1073/PNAS.1210595110/SUPPL_FILE/PNAS.201210595SI.PDF.

S2. Freeman, O.E., Duguma, L.A., and Minang, P.A. (2015). Operationalizing the integrated landscape approach in practice. E&S *20*, art24. https://doi.org/10.5751/ES-07175-200124.

S3. Thaxton, M., Shames, S., and Scherr, S.J. (2017). Integrated Landscape Management: An approach to achive equitable and participatory sustainable development (United Nations Convention to Combat Desertification (UNCCD)).

S4. Milder, J.C., Hart, A.K., Dobie, P., Minai, J., and Zaleski, C. (2014). Integrated Landscape Initiatives for African Agriculture, Development, and Conservation: A Region-Wide Assessment. World Development *54*, 68–80. https://doi.org/10.1016/j.worlddev.2013.07.006.

S5. Plieninger, T., Muñoz-Rojas, J., Buck, L.E., and Scherr, S.J. (2020). Agroforestry for sustainable landscape management. Sustain Sci *15*, 1255–1266. https://doi.org/10.1007/s11625-020-00836-4.

S6. Sayer, J.A., Margules, C., Boedhihartono, A.K., Sunderland, T., Langston, J.D., Reed, J., Riggs, R., Buck, L.E., Campbell, B.M., Kusters, K., et al. (2017). Measuring the effectiveness of landscape approaches to conservation and development. Sustainability Science *12*, 465–476. https://doi.org/10.1007/S11625-016-0415-Z/FIGURES/2.

S7. Reed, J., Van Vianen, J., Deakin, E.L., Barlow, J., and Sunderland, T. (2016). Integrated landscape approaches to managing social and environmental issues in the

tropics: learning from the past to guide the future. Global Change Biology *22*, 2540–2554. https://doi.org/10.1111/gcb.13284.

Supplementary Material: Detailed Initiative Descriptions

This supplementary material provides structured descriptions of the ten wildfire mitigation initiatives evaluated in the manuscript. The descriptions follow the five dimensions and associated factors of integrated landscape approaches used in the analysis.

1. MOSAICO (Spain)

Location: geographical boundaries and geographical and ecological features

Project Mosaico is being carried out in two adjacent but contrasting regions of northern Cáceres province, central-western Spain (see Map). The western region, Sierra de Gata (SG), is located at the border between Spain and Portugal and includes 18 municipalities encompassing 111,024 ha. The eastern region, Las Hurdes (LH), occupies 46,268 ha in five municipalities. Both are mid-elevation (500-1500 m a. s. l.) mountain regions under subhumid Mediterranean climate. In both regions forestlands (forest, shrubland and grassland) are the dominant land use, with 91.7% in SG and 81.0% in LH. In these areas shrublands resulting from secondary succession are the main vegetation type (43.8%) because of high fire recurrence in the last decades. Oak forests including Quercus pyrenaica, Q. suber and Q. ilex cover 15.2%, whereas pine (Pinus pinaster) plantations and secondary pine forests are present in 31.1% of the area. The remaining 9.9% corresponds to tree crops, especially olive (Olea europaea). Minor tree crops include chestnut (Castanea sativa), fig (Ficus carica) and cherry (Prunus avium). Tree crops are relevant productive fuel breaks. The study area harbours a total population of 17,675 inhabitants (13,040 in SG and 4,635 in LH). Global population density is 11.9 per square kilometre, which is under the threshold used to define Sparsely Populated Areas in Europe. Population has declined by 41.4% and 48.4% since 1960 in SG and LH, respectively. This depopulation process is still on-going and it is responsible for the drastic landscape changes observed in the last decades. Nowadays, most inhabitants (55.5%) work in the touristic sector or other kind of services.

What is it about? Main objectives

Mosaico is a large-scale, long term project aimed to gradually generate a Fire Smart Territory by engaging all types of local land managers from the agricultural, livestock and forestry sectors.Fuel load reduction through participatory landscape change. Two types of landscape interventions are developed to achieve this main goal: a) Indirect (non-strategic) prevention: landscape changes whose location is not related to the known distribution of main fire trajectories. b) Direct (strategic) prevention: targeted landscape changes located in relation to main fire trajectories.

Land use change tackled

The project combats forest and shrubland encroachment due to depopulation

Predominant land use dynamics

Forest transition (from agricultural to forest land) and extensification of forest management (less management forest lands). In both regions forestlands (forest, shrubland and grassland) are the dominant land use, with 91.7% in SG and 81.0% in LH (see Map). In these areas shrublands resulting from secondary succession are the main vegetation type (43.8%) because of high fire recurrence in the last decades. Oak forests including Quercus pyrenaica, Q. suber and Q. ilex cover 15.2%, whereas pine (Pinus pinaster) plantations and secondary pine forests are present in 31.1% of the area. The remaining 9.9% corresponds to tree

crops, especially olive (Olea europaea). Minor tree crops include chestnut (Castanea sativa), fig (Ficus carica) and cherry (Prunus avium). Tree crops are relevant productive fuel breaks

Main entry point /common concern

The big fire occurring in August 2015 in Sierra de Gata (central Spain). After the catastrophe there was a general consensus in that the underlying reason for such a fire was rural abandonment, loss of traditional farming practices and the subsequent spread of woody vegetation. Accordingly, our research team from Universidad de Extremadura (UEX) proposed a participatory initiative (project Mosaico) aimed to gradually reverse the trend by promoting agroforestry practices of preventive value. The proposal was approved in 2016 and became operational in September 2016. In both regions fires over 100 ha in area have been frequent, with catastrophic events burning over 3000 ha occurring in 1991, 2003, 2009, 2015, and 2022. According to official wildfire figures, the main causes of ignition are intentional fires (49%) and negligence (16%). Typically, shrublands and pine plantations (which in many cases have a dense shrub layer) are the most affected vegetation types.

ILA Dimensions

ILA Factors

MULTIFUNCTIONALITY

Multiple objectives

Profitable agricultural activities, indirect wildfire prevention (side positive effects: reduce depopulation, increase biodiversity). Agriculture, agroforestry, grazing, tree cropping, resing tapping, apiculture, eco-tourism. The project also develops continuous policy reporting and meetings at regional and EU levels.

Practices undertaken

Agriculture (mean area: 10.0 ha) entailed tree or shrub plantations (chestnut, olive, cherry, berries and aromatics) on bare soil which is tilled annually. Livestock (mean area: 208 ha) initiatives involved mainly goats and sheep either in single- or mixed-species herding. Finally, the forestry category (mean area: 31.9 ha) mostly included pine tappers (85% of interventions) followed by timber operations or two forest tree plantations using mainly tree oak species. A certification program has also been developed to provide a market label to increase sales.

TRADE-OFFS

Accounting for synergies and trade-offs

Synergies expected and actively searched for between productive extensive agroforestry, agricuture and forestry and widlfire risk mitigation

CROSS-SECTORAL

Multiple sector integration

The project address both land-use sectors (agriculture, livestock and forestry) and product processing and labelling, therefore engaging actors from the whole value chain.

Integrated management planning

Since 2016, the Mosaico base document is an agreement between the Directorate General of Forest Policy and the University of Extremadura, which will also be joined in 2025 by the Directorate General of Fire Prevention and Extinction. In 2022-2024 there was no agreement but the project has been maintained with European projects. As for spatial planning, there is no map with the final objective and all possible initiatives that contribute to the reduction of fuel and risk are simply developed. As for spatial planning, there is no map with the final objective and all possible initiatives that contribute to the reduction of fuel and risk are simply developed. The aim is for landscape transformation initiatives to coincide in space with previously located strategic areas against fires, although those that do not coincide are also developed.

PARTICIPATION

Transdisciplinary approach

Local stakeholders participated in planning and directly develop their proposals. Advice by the University and hired technicians. Monitoring by UNEX of performance in terms of imrpoved resilience against wildfires. Technical and traditional knowledge hibridation?

Role

Local stakeholders participated in planning and directly develop their proposals. Advice by the Univeristy and hired tehcnicians. Monitoring by UNEX of performance in terms of improved resilience against wildfires.

Degree of participation

To get these actors involved in the project we conducted, from October 2016 to June 2017, a public process explaining the goals of the project through focus groups in all the municipalities. During these sessions we encouraged the participants to join the project by sending their initiatives through the project's website. Proposals received were accepted for further support if they could achieve any degree of fuel reduction through the land use practices described above. After acceptance, initiatives were included in a database with their main attributes (type, area managed, goal, relevant skills and contact data of the person/association/institutionMosaico is a large-scale, long term project aimed to gradually generate a FST by engaging all types of LLM from the agricultural, livestock and forestry sectors. To get these actors involved in the project we conducted, from October 2016 to June 2017, a public process explaining the goals of the project through focus groups in all the municipalities. During these sessions we encouraged the participants to join the project by sending their initiatives through the project's website. Proposals received were accepted for further support if they could achieve any degree of fuel reduction through the land use

practices described above. After acceptance, initiatives were included in a database with their main attributes (type, area managed, goal, relevant skills and contact data of the person/association/institution in charge of the proposal). The information on initiatives was gathered in one or more face to face interviews by an expert technician from our team (a forest engineer, an agricultural engineer or a veterinarian depending on the type of initiative). The same technicians monitored and supported every single initiative from the onset to final execution or resignation. Visual examples are available in the project channel: https://vimeo.com/user58436665. Local stakeholders participated in planning and directly develop their proposals. Advice by the Univeristy and hired tehcnicians. Monitoring by UNEX of performance in terms of imrpoved resilience against wildfires.

ADAPTIVE MANAGEMENT

Adaptive

Since the onset of the project in 2016, the methodology has been updated after periodical reporting of results. Briefly, through the period 2016-2024, the size of the productive fuel breaks inmplemented has increased due to the association of producers and inreased financial support.

Performance

Measured as social-achievements, i..e nº of proposals and spatial distribution of interventions: whole set of proposals (n=250), 124 (49.6%) came from agriculturalists, 57 (22.8%) from forest producers, and 69 (27.6%) from shepherds. Regarding the success of the proposals presented, 94 (37.6%) had been implemented by the end of the study period. The rest of the initiatives (62.4%) were considered as non-implemented, with 43.6% deemed as failed and 18.8% in progress or with insufficient information. Fire-related achievements: We develop a simple (and transposable) method to assess the impact of initiatives on the fuelscape and thus fire spread potential. Briefly, we compared initial (before) and final (after intervention) fuel models for every single initiative. As an estimate of project performance we used the reduction in (simulated) burn area as a result of interventions. The table below shows the estimated impact for interventions made by LLMs (Impact S0-S1; mean: 10.54% reduction in burned area)

Funding

2016-2019 Funded by Junta de Extremadura plus competitive project funds of Universidad de Extremadura. 2020-2021 Funded by Junta de Extremadura plus competitive project funds of Universidad de Extremadura. 2022-2023 Funded through competitive project funds of Universidad de Extremadura.

WILDFIRE MANAGEMENT PHASES

Risk mitigation

Agriculture (mean area of productive fuel breaks, PFB: 10.0 ha) entailed tree or shrub plantations (chestnut, olive, cherry, berries and aromatics) on bare soil which is tilled annually. Livestock (mean area of PFB: 208 ha) initiatives involved mainly goats and sheep

either in single- or mixed-species herding. Finally, the forestry category (mean area of PFB: 31.9 ha) mostly included pine tappers (85% of interventions) followed by timber operations or two forest tree plantations using mainly tree oak species.

Readiness

The initiative does not address readiness actions

Recovery

the project unfolds in some areas previously burned)

2. LIFE GARNATHA (Italy)

Location: geographical boundaries and geographical and ecological features

Project Granatha is being carried out in the Pratomagno massif, located in the Tuscany region of central Italy. Pratomagno is a mountain range (500-1600 m) with a subhumid Mediterranean climate. The area covers 21,050 hectares and includes 12 municipalities with a total population of 35,681 inhabitants. It also encompasses the Natura 2000 site IT5180011, aimed at improving the conservation status of bird species listed in Annex I of Directive 2009/147/EC, which are associated with mountain heathland habitats, particularly Erica scoparia. The Pratomagno area is predominantly covered by broadleaf forests (65%), including beech woods, oak groves, and chestnut orchards. Shrublands occupy 10%, summit grasslands cover 7%, and reforestation areas with species like silver fir, black pine, and Douglas fir account for 5%. Mixed beech and silver fir forests also make up 5%, while agricultural land constitutes 4%. The remaining 4% consists of water bodies, rocky habitats, urban areas, and other artificial spaces. Due to the abandonment of traditional management activities (heather cultivation, forest coppicing, sheep grazing), these environments are transitioning toward older shrublands and forests, increasing wildfire hazards, especially at the wildland-urban interface. Additionally, these transformations are leading to the loss of breeding habitats for bird species of conservation interest.

What is it about? Main objectives

Granatha started as a European LIFE+ project (2017-2023) with the aim to reduce the loss of suitable habitat for target birds (Directive 2009/147/EC) in the Pratomagno area as a consequence of land use abandonment. Since the area is historically exposed to large wildfires, interventions to renew the heathlands have been planned integrating wildfire risk reduction with nature conservation goals. This means that part of the interventions for habitat maintenance are carried out along fuelbreaks and in critical nodes identified by the Strategic Wildfire Prevention Plan as key areas to be treated to increase fire suppression capacity and security. Furthermore, since the area is a mix of private and public lands, the initiative involves local private owners and municipalities providing lands. The intervention areas consist of 27.6% privately owned land, while the remaining 72.4% is publicly owned and falls within the regional domain of Tuscany. Some of the privately owned areas were not managed for over 30 years. The project mobilized more than 30 private owners that have provided their properties for the Plan implementation. In order to create a sustainable management involving both private and public actors, the Granatha included among its goals the creation of a value chain to produce and commercialize organic/biological brooms made of Ericaceae by using vegetation residues cut along fuelbreaks and nature conservation areas. The project aims raising awareness in the public of the role of nature conservation in wildfire risk management and the importance of maintaining managed agro-forestry landscapes for fire hazard abatement.

Land use change tackled

Loss of profitability, degradation of Habitats (Natura 2000) key for bird conservation, and increase in lanscape flammability.

Predominant land use dynamics

Forest trasition (from agricultural to forest land) and extensification of forest management (less management forest lands). The Pratomagno area is shifting towards aged shrublands and forests due to the abandonment of traditional management activities, leading to a general increase in wildfire hazard particularly at the wildland urban interface. These transformations result in the loss of breeding habitats for bird species of conservation interest, including Sylvia undata, Lullula arborea, Lanius collurio, Circus pygargus, Anthus campestris, and Caprimulgus europaeus.

Main entry point /common concern

The Granatha project stems from the need to recover active agro-forestry-pastoral land management in the Pratomagno area. The abandonment of active forest management and production of Erica biological brooms present in the 20th century until the 1970s has resulted in a loss of open habitats suitable for European protected bird species and has increased fire hazard at the landscape scale with the occurrence of relatively large wildfires in recent decades.

ILA Dimensions

ILA Factors

MULTIFUNCTIONALITY

Multiple objectives

The Granatha project aims to reduce the loss of suitable habitat for target birds (Directive 2009/147/EC). However, since the area is historically vulnerable to large wildfires, interventions to renew the heathlands have been planned integrating wildfire risk reduction with nature conservation goals. This means that part of the interventions for habitat maintenance by mechanical cutting and prescribed burning are carried out along productive fuelbreaks and in critical nodes identified by the Strategic Wildfire Prevention Plan as key areas to be treated to increase fire suppression capacity and security. Moreover, the Granatha created a value chain to produce and commercialize organic/biological brooms made of Ericaceae by using vegetation residues cut along productive fuelbreaks and nature conservation areas. Meanwhile, prescribed burning interventions are integrated in the training program to the use of fire of the Toscana Region. The project aims raising awareness in the public of the role of nature conservation in wildfire risk management and the importance of maintaining managed agro-forestry landscapes for fire hazard abatement.

Practices undertaken

Fuel management by mechanical treatments and prescribed burning whose spatial distribution accounts for both strategic fire hazard reduction, intervention costs abatement and habitat conservation for avian species included in EU Directive 2009/147 EC. Interventions goals are to reduce flammable fuels along fuel breaks and in blocks, restore habitat for a number of target avian species by reducing tree encroachment and increasing shrubland structural diversity at both the stand and landscape scale, extract valuable material for the production of bio-brooms destined to Urban cleaning services.

TRADE-OFFS

Accounting for synergies and trade-offs

Synergies actively searched for between wildfire risk mitigation and recovering pastoralism and shrub clearing for broom production. Habitat conservation for key bird species whose habitats are threatened by vegetation encroachment. Interventions carried out in LifeGranatha contribute to fire prevention in three different ways: i) reduce landscape scale flammability by biomass removal in strategic areas increasing fire-fighting operation security and effectiveness in the area; ii) prescribed burning interventions are carried out in collaboration with the "Regione Toscana prescribed burning training program" thus contributing to increase expertise among fire professionals; iii) the value chain of bio-brooms production increases economical interest on the area with indirect effect on land control and care with positive cascading effects on wildfire prevention.

CROSS-SECTORAL

Multiple sector integration

The initiative involves different sectors of the Fire Management System of the Regione Toscana including: i) forestry sector; ii) civil protection; iii) fire-fighter regional training school; iv) local land management agency (Unione Comuni, representing local municipalities) with competences on wildfire management; v) volounteers fire brigades. Moreover, the initiative involved the Nature Conservation sector of the Regione Toscana which has competences on assessing fire effects on target habitats. National agencies with wildfire related competences supporting the Regione Toscana with both fire fighting (Corpo Nazionale Vigili del Fuoco) and fire cause investigation (Carabinieri Forestali) were informed and involved in the LifeGranatha prescribed burning program and Strategi Wildfire Prevention Plan.

Integrated management planning

The Strategic Wildfire Risk Mitigation Plan of the area integrates treatment areas for habitat 4030 conservation, productive fuelbreaks for biological brooms production, and forest management areas for wood production. To create a local supply chain of organic brooms ensuring continuous and sustainable management of heathlands, economic actors were involved, and a marketing plan including productive fuelbreaks was elaborated to understand the market price of organic brooms, the target of the marketing and the added value of wildfire prevention as an ecosystem service in the final commercial price.

PARTICIPATION

Transdisciplinary approach

Knowledge from diverse spheres and sectors, including policy makers, land managers, researchers, and local actors with historical knowledge, is effectively integrated in the management and execution of the nature conservation and wildfire mitigation plan. The Granatha project was politically supported by the Tuscany Region which facilitated administrative processes (e.g. Plan authorization), the engagement of key stakeholders (e.g. national agencies), and communication to citizens (e.g. explaining wildfire mitigation strategies such as prescribed burning). Forest technicians from the Public Land Management Agency (Unione Comuni del Pratomagno), contributed with technical knowledge developing planning tools and implementing wildfire risk mitigation on both private and public land through vegetation management and prescribed burning techniques, coordinating forest workers. Local actors with historical knowledge provided and transferred to NGOs the technical and ecological expertise to set the broom production value chain. Scientific knowledge from the University of Torino was key to develop the management plan and monitored intervention areas during the program's first five years and the After Life phase, ensuring an evidence-based approach and indicators for adaptive management. The private company DREAm Italia provided the managerial knowledge to coordinate the program.

Role

The enterprise DREAm Italia coordinated the project; Union of municipalities (Unione dei Comuni - land management agency) developed and implemented the nature conservation and wildfire risk mitigation plan and signed a formal agreement of 20 years with private onwers; private owners provided lands; NGOs did the business plan created the values chain for brrom production; Regional administration provided political support, regulation, and authoriaztion processes; national fire management agencies (Vigili del Fuoco and Carabinieri Forestali) controlled the Plan implementation; private enterprises carried out most of cutting interventions; volunteer associations supported prescribed burning treatments; research agencies carried out the ecological monitoring.

Degree of participation

Phase 1 - project buiding: Private enterprise DREAm as promoter of the project and coordination of project partners (Unipone Comuni, Universities, Regional Fire Management System, NGOs) and contacting private owners. Phase 2 - Planning: Unione dei Comuni; Regional Fire Management Agencies and University collaborates to design the Wildfire Risk Mitigation Plan. Phase 3 - Project implementation: Unione Comuni coordinates local enterprises to carry out cutting intervention. The Regional Fire Management Agencies coordinates the prescribed burning program. Phase 4 - Value chain creation: DREAm and NGOs design the business plan and create the association that commercialize organic brooms. Phase 5 - Monitoring: Universities and professionals monitor treatment effects on vegetation, biological indicators (eg. carabidae) and birds communities. Phase 6 - Plan

adaptation: mid-term assessment of initiative results after 5 years with all partners involved.

ADAPTIVE MANAGEMENT

Adaptive

Since the initiative began as a 6-year LIFE project, continuous monitoring with success indicators is a structural element of the Granatha project. Every year, at least two project assessment meeting have been held among all the involved stakeholders to adapt the project to emerging needs based on environmental and socio-economic indicators. Upon the closure of the LIFE project, a careful evaluation was conducted on the objectives achieved and the limiting factors that required adaptation measures in the currently ongoing AfterLIFE program.

Performance

The project adopts fuel reduction, ecological and economical indicators which have been monitored since 2017. These includes: (i) total amount of area managed by vegetation treatments; (ii) abundance and diversity of target bird species; (iii) treee basal area reduction and schrubland cover increase; (iv) biological indicators such as abundance and diversity of insects, with a particular focus on the Carabidae family; (v) number of contracts to sell biological brooms; (vi) wildfire burnt area within the Natura 2000 boundaries. Fuel reduction indicators are assessed at both stand and landscape scale: i) stand: shrub cover and height reduction; ii) landscape: percentage of area treated; iii) width of fuel breaks.

Funding

Starting: LIFE program. Prescribed burning: funding from the regional firefighting budget (framed as training). To establish a sustainable local supply chain for organic brooms and ensure ongoing management of heathlands, economic stakeholders were engaged, and a marketing strategy was developed. This strategy aimed to determine the market price of organic brooms and identify the appropriate target audience for marketing efforts.

WILDFIRE MANAGEMENT PHASES

Risk mitigation

Prescribed burning is carried out in natural areas with difficult access for mechanical means, making economic activities unfeasible. These areas, important for various bird species, exhibit diverse environments with open spaces, shrublands, and small woodlands, and do not require constant maintenance due to their vegetation dynamics. Clearing activities are tailored to accessibility and the area's purpose. Productive areas, with easier access and denser heathland, are prioritized for harvesting Erica scoparia for broom production. Natural areas, with more challenging terrain and lower Erica presence, support bird species and require less frequent management. Intervention types include mechanized and manual cutting methods, either clear or in patches, based on terrain and objectives. Prescribed burning, incurs relatively low costs ranging from €400 to €1,000 per hectare. For mechanical and manual clearing, based on Tuscany's regional forestry price list and

adjusted for mountainous terrain, costs are estimated at €1,325.52 for mechanized cutting and €2,200.50 for manual cutting per hectare. Including temporary service roads and transport of cut vegetation, the total average cost per hectare is around €3,100.

Readiness

The Granatha project has integrated the entire prescribed fire plan for the maintenance of firebreaks and habitat conservation into the fire training program of the Tuscany Region. Each prescribed fire intervention under Granatha has involved regional firefighters from across the region to deepen their fire usage techniques on Granatha sites and enhance the overall readiness of the regional wildfire prevention system. These operators must acquire training credits on prescribed fire sites to obtain certification as fire analysts and backfire operators.

Recovery

The initiative does not address recovery actions

3. RAPCA (Spain)

Location: geographical boundaries and geographical and ecological features

The network of grazed fuelbreaks of Andalusia is located in public woodlands in the southernmost region of Spain. During the year 2022, 169 contracts with shepherds have been signed, of which 123 correspond to contracts carried out on mountains owned by the Junta de Andalucía and 46 on woodlands owned by local councils. The area grazed by livestock (mainly sheep and goats) in the network of fire-break grassland areas of Andalusia (RAPCA) reached 5,433.20 ha. The budget allocated was: 892.193,39 €.

What is it about? Main objectives

Its main objective is to use targeted grazing in fuel breaks in public forests in the region to control biomass and reduce the costs of mechanical clearning in these strategic infraestructures

Land use change tackled

No land use tackled. The forest land is still forest land with the targeted grazing program.

Predominant land use dynamics

Increase in forest cover, limited investment in forest management due to reduced profitability. Reduction of traditional activities, included extensive grazing.

Main entry point /common concern

Reducing the costs of using heavy machinery and have a more regular maintenance of fuelbreaks.

ILA Dimensions

ILA Factors

MULTIFUNCTIONALITY

Multiple objectives

The main objective of the RAPCA is reducing the costs of mechanical biomass reduction. A second objective is the integration of traditional activities, such as extensive grazing into the wildfire mitigation activities and the reduction of historical conflictual relationships between foresters and graziers.

Practices undertaken

Extensive graziers undertake controlled grazing activities reducing biomass in fuelbreaks in public forests. These fuelbreaks should be previously cleared from biomass and then the shepherds control the regrowth of the vegetation. Controlled grazing performance is monitored by RAPCA technicians and payments for the biomass reduction service is provided acordingto the level of difficulty and accomplishment of the network

TRADE-OFFS

Accounting for synergies and trade-offs

Trade-offs and synergies are not explicitly considered between standard mechanical wildfire prevention and controlled grazing in the sense that coordination between both activities is not completely achieved. The linkage of the RAPCA scheme to the lease contracts for the shepherds to access pastures in public forests. Where the lease area granted to a shepherd encompasses fire breaks, maintenance of fire breaks may be entrusted to that shepherd within RAPCA. However, the shepherd does not receive a direct payment for the fire prevention service provided, but rather experiences a reduction in the annual lease price for the equivalent amount. Nowadays, both options, either a RAPCA contract or a contract linked to the lease are in place. The merging of lease contracts with the PES scheme may distort the philosophy of the payment mechanism. Shepherds' role changes from being providers of a risk reduction service to the administration (highly valued, positive behavioural reinforcement) to becoming the potentially bad agent, e.g., considering reduced biomass reduction targets as a punishment ("I have to pay more for the lease if I do not perform well")

CROSS-SECTORAL

Multiple sector integration

The regional agency of environment and water (AMAYA) manages RAPCA: it handles the contracts with the shepherds. The RAPCA technicians belong to AMAY and undertake most of the coordination work involving the regional and provincial services of wildfire prevention and supression (i.e. the regional INFOCA program), local councils and agricuktura offcie networks. The program does not integrate as such the livestock sector, either regional livestock farming unions, regional agrarian offices or the agriculture department. It does not integrate either conservation/environmental departments. There is not an explicit aim for the integration of other sectors beyond wildfire services (INFOCA) and the regional agency that manages the contractual relationship with farmers (AMAYA).

Integrated management planning

RAPCA does nos show an intention to advocate for further integration, existing a lack of coordination between the mechanized and manual clearing of fuelbreaks and the clearing needs fo the RAPCA for controlled grazing to be sucessful. As such RAPCA thanks to its technicians rather adapts to the clearing plans of INFOCA, replacing excessively scrubbed areas with recently cleared ones by mechanical means.

PARTICIPATION

Transdisciplinary approach

The transdisciplinary approach was shown in its design and initial steps where scientific knowledge on pastoralism was blended with technical knowledge by foresters that could indicate areas where targeted grazing would be most needed while traditional pastoral knowledge was required to develop the program. The current development of the program

does not show such blending with a more top-down approach where the wildfire management program (INFOCA) and the regional agency issuing the contracts (AMAYA) manage RAPCA with no/little intersectoral communications. RAPCA technicians undertake these interactions in a non-formalized and unofficial way.

Role

The RAPCA fuel breaks are located in public forests of Andalusia, with a management plan supervised by a forestry engineer belonging to the Forest Service, and are surveilled by forest guards. Hence, the involvement and support of the Forest Service is essential for the inclusion of a given forest in the scheme. Often these forests are located in regional Natural Parks that have their own regulations and management plan. Fire prevention activities in these forests are undertaken by INFOCA staff and include for example mechanical clearance of biomass in fuel breaks or tree pruning. These actions are decided by COP and COR offices in agreement with the forest service or the head of the Natural Park. Coordination between all these agents is needed for granting shepherds access to the forest areas that were not previously grazed and wherein improvement of pastoral infrastructures may be needed. The RAPCA staff deploys horizontal and vertical coordination efforts to fit the RAPCA scheme into the existing institutional framework. The initiative in its management includes actors with power and interest.

Degree of participation

In its current format RAPCA entails a low degree of participation, notably of the shepherds that provide the controlled grazing services, who are only informed but not consulted about its development. RAPCA technicinas do establish a dialogue with shepherds to achieve optimal performance, but there is no formalized protocol for these interactions that henec depend on the good will of both sides. Trust in tehcnicinas is however key for shepherds to engage in the proram, since participation is voluntary and the most suitable shepherds are invited to participate in RAPCA.

ADAPTIVE MANAGEMENT

Adaptive

The initiative has not followed any type of evaluation and remains unchanged since its deployment in 2010. This means that the coordination defficiencies with mechanical browsing are not dealt with. The payments to the shepherds set up in 2010 that range between 42 and 90 €/ha depending on the level of difficulty remain unchanged and hence very low, specially when compared with other regions in Spain that have adopted similar schemes following RAPCA example.

Performance

Some scientific studies have looked in a very preliminary way at avoided costs by controlled grazing when compared with mechanical means, but there is no evaluation of performance formally adopted by the administration, beyond accounting annually for the number of shepherds, hecatores covered and budget allocated to the measure.

Funding

Funding has been available for a long-term period, since 2010. The fact that RAPCA budget depends on wildfire prevention funds made it stable thorughout the last decades. However, its dependence on a single entity also makes it vulnerable to an eventual cancellation. In fact, the freezing of the payment levels to the shepherds since 2010 and the glaring lack of will to undertake performance checks or involving the shepherds seem to point towards a decay of the program.

WILDFIRE MANAGEMENT PHASES

Risk mitigation

The initiative focuses on risk mitigation on fuelbreaks whiel nearby areas are also subject to grazing and hence biomass reduction.

Readiness

The initiative does not tackle readiness actions

Recovery

The initiative does not tackle recovery actions

4. ADF Matadepera (Spain)

Location: geographical boundaries and geographical and ecological features

The initiative is located in the municipality of Matadepera (Catalonia, Spain). The municipality covers an area of 25.4 km2 and has a population of 9496 inhabitants (2020). Elevation range is ca. 400–1100 m.a.s.l. It is located on the hillside of the pre-coastal mountain ranges Sant Llorenç del Munt and l'Obac. 61% of the municipality belongs to the Natural Park Sant Llorenç del Munt and l'Obac. Matadepera is a wealthy residential suburb located in the second ring of the Barcelona Metropolitan Region, neighbouring the cities of Terrassa and Sabadell, two metropolitan sub-centres of more than 200,000 inhabitants each. It has an extensive low-density wildland-urban interface spanning between the old town centre and the natural park.

What is it about? Main objectives

The main objective is to build resilience to large forest fires by decreasing fuel load in strategic management areas through a land management scheme (strategic management areas are wildfire-friendly landscape structures that, if implemented, facilitate suppression and limit wildfire size). See other related objectives under Multifunctionality.

Land use change tackled

Agricultural abandonment, forest abandonment, urban sprawl.

Predominant land use dynamics

Urban land in the municipality increased from 38 ha in 1956 to 360 ha in 2008, while cropland declined from 228 ha to 42 ha in the same period. The rural municipality of the beginning of the 20th century, with a small compact centre surrounded by a ring of gardens, cereal fields, vineyards and olive trees separating it from the forests, became an extensive low-density wildland-urban interface. Land-use is today fundamentally residential and related to leisure outdoor activities (hiking, biking, etc.) in extensively forested hills.

Main entry point /common concern

The fire of Sant Llorenç Savall (2003) affected neighbouring municipalities (ca. 4600 ha). Many volunteers of Matadepera participated in the suppression tasks. It strongly affected people in Matadepera and put several of the volunteers at risk. In the aftermath of the fire, volunteers from ADF of Matadepera started working on a land management scheme to build resilience to this kind of events. Hence a large disturbance triggered social organization and learning to adapt, as predicted by the literature. However, structural conditions were there for this initiative to flourish: 1) GRAF (created in 1999) was increasingly influential in their approach to fire management, especially since their methods had proven useful in containing precisely that fire of Sant Llorenç Savall (which had a potential of tens of thousands of ha and only burnt 4600 ha). A local forest engineer from Matadepera working as a volunteer for ADF and working professionally in the Catalan forest administration, was close to GRAF's approach and networked with them to draft the Strategic Management Areas that would become the backbone of the land management scheme; 2) Matadepera is one of the Catalan municipalities that ranks higher in terms of socioeconomic development.

This means that the level of income and education is higher than the Catalan average. The board of ADF contains people holding detailed local knowledge and technical or scientific professions related to environmental management. In addition, one of the main assets of Matadepera is the high-quality landscape in the foothills of a Natural Park, which not only attracts real estate development but is also a distinctive feature related to local identity and pride, motivating volunteers to work with ADF and citizens to support ADF's work. 3) The fact that different institutions provided their funding for the development of the land management scheme was also a facilitating factor (see "Funding").

ILA Dimensions

ILA Factors

MULTIFUNCTIONALITY

Multiple objectives

The main objective is to build resilience to large forest fires by decreasing fuel load in strategic management areas. Other related objectives (which relative importance can vary with time, since this is an initiative spanning over ca. 20 years) are: to promote the social memory of the rural past; to enhance open habitats of cultural and conservation interest; to stop the encroachment of residential developments in the forests. These other objectives could be more or less explicit for different people involved in the initiative.

Practices undertaken

Multiple practices are undertaken: delimitation of Strategic Management Areas (SMA) by the Fire Department's expert group on wildfires (Grup de Recolzament d'Actuacions Forestals, GRAF); design of a land management scheme with interventions to reduce fuel load in SMA based on the maintenance of cropland, thinning/pruning of forests and extensive grazing (in ca. 140 ha); approval of land management scheme by Town Council; implementation of interventions through formal and informal agreements with farmers, forest owners, forest workers, shepherds and other actors; monitoring of interventions' results through observation and (short-term) scientific research; public outreach on wildfire risk and mitigation measures. All this was accompanied by: the maintenance of forest tracks and water infrastructures and by patrolling shifts during high-risk season (integrated in municipal and regional wildfire prevention schemes); actions to activate the social memory of the rural past (notably through historical research and outreach); actions to protect local landscape and rural heritage from urbanization. So far unsuccessful attempts to conduct prescribed burning.

TRADE-OFFS

Accounting for synergies and trade-offs

Synergies are considered between fire risk reduction activities and forestry, agriculture, grazing and related production and commercialization of local products. Synergies are also considered between implementation of strategic management areas and the recovery or

activation of the municipality's rural past, where the landscape mosaic of the beginning of the 20th century is taken as a reference point, not to go back to it but rather as a symbolic and practical inspiration. Trade-offs can exist between prescribed burning and the inhabitants of wildland-urban interface due to smoke disturbance. This was one of the reasons put forward by the Natural Park to deny authorisation for prescribed burns (the other being that the Natural Park's special plan does not allow this particular use of fire). Another potential trade-off is that building wildfire resilience can increase the attractiveness of the wildland-urban interface and bring new inhabitants, thus reinforcing current vulnerability patterns, but this remains an abstract consideration so far.

CROSS-SECTORAL

Multiple sector integration

The Catalan Fire Department's expert group on forest fires (Grup de Recolzament d'Actuacions Forestals, GRAF) provided technical support to delimit Strategic Management Areas. The land management scheme of ADF was approved by the Town Council thus involving the policy domain. Several municipal services also participated, for example when coordinating certain tasks (like the implementation of low fuel strips around urban areas) with the ADF scheme. The interventions foreseen by the scheme were implemented through formal and informal agreements with farmers, forest owners, forest workers, shepherds and other actors. The Natural Park of Sant Llorenç del Munt i l'Obac was likewise involved through the authorisation of most interventions (not all of them) and funding some of them. One private actor also became involved by funding some interventions through the creation of an ad hoc association. The products of the flock currently grazing in the municipality for wildfire risk reduction are sold in the shops of the region (meat). The Local Environmental Council was active in the activation of the rural memory through dissemination activities.

Integrated management planning

After the Sant Llorenç Savall wildfire of 2003, the Matadepera Forest Defence Group (Agrupació de Defensa Forestal, ADF) started working on a land management scheme aimed at building resilience to large forest fires. The scheme was officially approved by the Town Council in 2005 and subsequently started to be implemented. The implementation of this scheme has been the backbone of a process for building resilience that is still active as of 2024 (see previous rows for details on the sectors involved in such implementation). The plan is complemented by other initiatives (like grazing) that don't have a plan as such but are integrated in the objectives of the mentioned management scheme.

PARTICIPATION

Transdisciplinary approach

See information under Multiple sector integration. Several actors from the practice were involved. The policy domain was also involved through the funding or approval of initiatives by public administrations at municipal and regional (province, autonomous community) levels. Science participated through the involvement of the Autonomous University of

Barcelona (research on landscape change, political ecology of urbanization and monitoring of grazing effects on vegetation) and the Matadepera Archives (research on the environmental history of the municipality). In addition, we could say that ADF of Matadepera is a boundary spanning organization composed of representatives of the Town Council, the forest owners of the municipality and volunteers. However, research questions were not necessarily co-constructed between science, policy and practice. This is rather an initiative led by some community members that leverage their expert and local knowledge through a boundary spanning organization such as ADF.

Role

It depends how you define power and interest. Definitively actors with interest on wildfire management were actively involved (ADF, GRAF). If we consider that the Town Council is an actor with power since by law it has the main responsibility on wildfire prevention, then we can say that it was involved by approving or even funding some of the initiatives. This is important because de facto it implies that the Town Council transferred some of their responsibilities in wildfire prevention to ADF. Actors with even more power, such as those 'deciding' on the development model underlying wildfire risk (real estate agents, policymakers at larger scales, etc.) were not involved.

Degree of participation

The actors mentioned in the previous rows were consulted and participated to different degrees in the implementation of the management scheme, as explained above. In addition, citizens were also informed in different public events and articles in local newspaper, without necessarily a process of consultation but rather a one-way communication from initiative leaders to the citizens. However, the design of the management plan was mostly done by ADF and GRAF technicians.

ADAPTIVE MANAGEMENT

Adaptive

Evaluation and adaptation happened in a rather unstructured or emerging way. For example, after the denial of the Natural Park to authorize a prescribed burn around 2012, ADF planned another burning outside the borders of the natural park in order to showcase the benefits of this practice without depending on the natural park's favourable report. The burning has been recently authorized, in-situ delimited by the Fire Service, and is now awaiting the proper weather conditions to be conducted. Another example of adaptation is the inflow of private funding. When one of the landowners announced to ADF his will to privately fund some works to improve the natural heritage and prevent forest fires in the municipality, ADF was ready to suggest new strategic management areas (SMA) based on its land management scheme. These areas were hence implemented with the mentioned private funding. Visits to the SMA of Matadepera together with GRAF technicians and technicians from the Catalan Forest Property Centre were conducted later on to check if ADF SMA had an appropriate structure for suppression works and to identify additional necessary interventions. The technical criteria to implement SMA were adapted accordingly

and new SMA were added in the project's cartography. In addition, a joint management scheme with the neighbouring municipality of Terrassa was approved which added some additional SMA to the already existing SMA of ADF scheme.

Performance

There is no formal long-term monitoring of performance in terms of the overall effects of the land management scheme on the potential intensity of a forest fire. However, some initiatives partially substitute for this. Together with researchers from the Autonomous University of Barcelona, ADF managed to monitor the effects of grazing in the understorey for 2 years immediately after the introduction of the flock (around 2008 and 2009), indicating an incipient change of species composition and a certain (low) control on plant growth. In addition, as reported under Adaptation, visits to the strategic management areas (SMA) together with GRAF technicians and technicians from the Catalan Forest Property Centre were conducted later on to check if these SMA had an appropriate structure for suppression works and to identify additional necessary interventions. This amounts to checking the SMAs potential effects on fire intensity. Finally, ADF monitors the state of the Strategic Management Areas through observation on a continuous basis. Although there is no control against which to compare the effectiveness of interventions, it is likely that the municipality's resilience is higher than in a hypothetical non-intervention scenario, especially considering climate warming in the last decades.

Funding

The initiative was/is funded by different institutions including the Matadepera Town Council, the Catalan Department of the Environment, the Natural Park of Sant Llorenç del Munt and l'Obac, and private foundations. Funding from different sources has thus been available in a sustained way over the medium and long-term.

WILDFIRE MANAGEMENT PHASES

Risk mitigation

Several risk mitigation actions at different scales and targeting various risk causes were undertaken. In particular, actions tackled biomass reduction at the landscape scale to reduce the potential intensity of wildfire; biomass reduction at the (wildland-urban interface) garden scale to reduce consequences of fire for residential areas; the maintenance of forest tracks and water infrastructures to ease suppression works; patrolling shifts during high-risk season to prevent ignitions from spreading. See more information under "Practices undertaken".

Readiness

The initiative includes some readiness actions. For example, there is an evacuation plan approved by the Barcelona Province Authority providing information to all neighbours of the wildland-urban interface on evacuation plans and self-protection measures. Even if this was not done by ADF, ADF later added some efforts in this direction. First, a study of the state of fuels in private gardens belonging to the wildland-urban interface was conducted, leading to a prioritization of gardens, suggested fuel-reduction actions and active requests

to owners to engage in such actions. Second, meetings were held with associations of neighbours (normally each residential development has its own association of neighbours) before the start of the fire season, where the risk situation/forecast for that season was presented, as well as the measures undertaken by ADF to address fire risk. These workshops were also used to provide information on options that houses in the wildland-urban interface have to improve their gardens, including self-protection measures and recommended behaviour in case of fire. In these meetings, some neighbours proactively asked ADF to visit their houses to concretize the recommendations. In addition to all this, ADF collaborates with local newspapers in outreach related to fire risk and mitigation measures. Finally, the volunteers of ADF and the local fire service are regularly trained on fire suppression measures, normally by the Catalan Fire Department' School.

Recovery

The initiative does not address recovery actions.

5. RAMATS DE FOC (Spain)

Location: geographical boundaries and geographical and ecological features

This initiative is being implemented in Catalonia (north-east Spain), where the landscape has undergone dramatic change in the last 70 years due to rural abandonment and the loss of economic activity. The reduction in traditional land uses, mainly agriculture, extensive livestock farming, and multipurpose forestry (e.g., timber, wood fuel, charcoal, resins), has allowed for the regeneration of secondary vegetation, characterized by the proliferation of shrubby and bushy species. At the same time, the landscape has also suffered a process of homogenization, with thousands of hectares forming continuous masses of young and unstructured fire-prone forests. In this context,

What is it about? Main objectives

The project promotes silvopastoralism to graze Mediterranean mixed forests (Aleppo pine, Holm oak, Cork oak...) and understory vegetation (Erica sp., Cistus sp., Quercus sp....) decreasing fuel loads in areas where the fire services foresees a change on fire behaviour and suppression opportunities. The main impacts that the project wants to achieve are threefold:

- To reduce the risk of large wildfires through prescribed grazing (predefined management objectives to achieve defined goals). In general, the project's livestock farmers graze strategic fire management areas (SMAs) defined by the Fire and Rescue Service.
- To value the work done by the shepherds and the flocks as an ecosystem service (wildfire prevention, biodiversity, etc) through a payments for ecosystem services in agreement the Catalan goverment. Thus, the govenrment is also involved by certified the treatment done in the SMA.
- To promote selling of the products through the use of the FireFlocks label in restuarants and butchers, as away to enhance visibility of the project and raise awarness to the general population about 1) wildfire risk reduction, 2) the need to consume local productsto support the shepherds and revert rural abandonment.

Land use change tackled

Regeneration of secondary vegetation, characterized by the proliferation of shrubby and bushy species due to land abandonment

Predominant land use dynamics

During the second half of the XX century, Catalonia experienced significant landscape dynamics caused by rural abandonment and rapid urbanization. Socioeconomic shifts made people move from rural areas to large cities, seeking better economic opportunities. This migration led to rural depopulation, leaving traditional agricultural lands and pastoral systems abandoned.

These lands, previously managed through forestry, farming and grazing, underwent natural succession, resulting in dense, unmanaged forests and shrublands. The traditional mosaic landscape of mixed uses transitioned to homogenous forest stands, increasing wildfire risks due to higher fuel loads.

Simultaneously, Catalonia's urbanization surged, driven by industrialization and tourism. Urban sprawl expanded into peri-urban and previously rural areas, transforming them into residential, commercial, and industrial areas.
These changes disrupted the ecological balance and posed environmental vulnerabilities. The loss of traditional land management practices and the expansion of urban areas reshaped Catalonia's landscapes.

Main entry point /common concern

The Fire Flocks initiative emerged due to increasing wildfire risks in Catalonia, triggered by rural abandonment since the 1960s, leading to dense, young forests with high fuel loads. The Mediterranean climate's hot, dry summers exacerbated this risk. Conditions allowing its emergence included regional awareness of fire hazards, support from local (shepherds, landowners, local councils) and regional (fire service, butchers association, Climate Action Department) stakeholders, and the need for sustainable land management. Silvopastoralism, integrating livestock grazing to manage understory, was recognized as an effective solution to create safer, more manageable forest areas, reducing wildfire threats and supporting rural economies.

ILA Dimensions

ILA Factors

MULTIFUNCTIONALITY

Multiple objectives

(1) Implement and monitor grazing in Strategic Management Areas (SMAs) to reduce fuel loads effectively; (2) Enhance the market value and demand for products derived from extensive livestock farming through certification and labeling; (3) To engage new shepherds to the project providing financial incentives and support for grazing in SMAs; (4) Facilitate partnerships with local butchers and restaurants to create a value chain for bioeconomy products, including meat and dairy products that each sheperd comercialises with their local network; (5) To represent the extensive livestock sector in the policy arena to achieve improvements in the sector, for instance (i) support the public administration on the definition of a system of payments for ecosystem services. Thus, the farmers and sheperds received a more just compensation for the services provided to the society (i.e. wildfire prevention, management of the forest, enhancing biodiversity). (ii) Carry out pilot sites, and analyse the pontential that a payment system like this would have in the whole Catalonia area (beyond the Fireflocks project).

Practices undertaken

(1) Engagement of 72 farmers and sheperds to graze in SMAs, first in the region of Girona and then expanding to other areas of Catalonia.
(2) Promotion of new distribution channels in the case of generating agri-food products, this includes the direct comercialisation, distribution to restaruants butcheries, etc. Selling all the products through the Fireflocks label to promote a brand-positioning strategy while

raising risk awarness.
(3) Involvement of the competent administrations in the governance of the project. They are able to support in mapping the strategic management areas, establish the criteria for payment for services, simplification and adaptation of the legislative and administrative bureaucracy to the reality of the sector;
(4) Support to the establishment of new livestock projects aimed at grazing to manage strategic areas, increasing the current impact of the sector on the management of the forest space;
(5) Awareness raisong of pastoralism's impacts to society and transfer the message to a large part of society through dissemination, awareness and education; For instance, Fireflocks has been collaborating for two years with Primavera Sound music festival to provide meat of the project to VIP attendees, thus raising awarness to target groups usually difficult to reach: young, urban population and turists.
(6) Promoting a fair payment for managing the vegetation in SMAs and involving the public administration. Influence public policies that affect extensive livestock farming, specifically focused on fire risk management, for the defence of the values of human activities and ecological processes that favour the maintenance, conservation and sustainable management of the mosaic landscape.

TRADE-OFFS

Accounting for synergies and trade-offs

Grazing SMAs reduces fuel loads, directly lowering wildfire risks and creating economic opportunities for farmers. By promoting grazing in these areas, the initiative effectively integrates fire prevention with livestock productivity. Additionally, valorizing the shepherd profession and enhancing the market value of livestock products derived from extensive farming increase farmer incomes.

CROSS-SECTORAL

Multiple sector integration

The initiative involves multiple sectors related to wildfires. Emergency services identify SMA critical for fire prevention. Foresters collaborate to promote forest management practices, preparing these lands for grazing. Extensive livestock farmers then graze these areas to reduce fuel loads. Additionally, the Climate Action Department supports the initiative by promoting subsidies and payments for wildfire prevention services.

Integrated management planning

The project is guided by a strategic document developed in collaboration with various stakeholders, including shepherds, fire services and the Climate Action Department, among others. This document addresses key topics such as the Strategic Management Areas (SMAs) where the project operates and the detailed implementation methodology. It also outlines the use of the FireFlocks label and certification processes to ensure quality and sustainability. The roles and responsibilities of each stakeholder are clearly defined,

ensuring coordinated efforts in wildfire prevention, land management, and economic promotion.

PARTICIPATION

Transdisciplinary approach

The initiative employs a transdisciplinary approach, integrating knowledge and expertise from various fields to address wildfire prevention comprehensively. By involving shepherds, fire services, foresters and the Climate Action Department, the project combines practical agricultural practices with advanced forest management and emergency planning.

Role

Shepherds graze wildfire strategic areas.
Landowners sign agreements with shepherds in order to let them graze their properties.
Catalan Fire and Rescue Service identifies the wildfire strategic areas according to risk scenarios based on historical wildfire events and future projections.
Catalan Climate Action Department promotes an annual subsidy since 2018 for fire prevention grazing. The payment depends on two variables: managed hectares and the category of those hectares in terms of fire prevention.
Local butchers and restaurants are leading the valorisation strategy of livestock products. They use the Fireflocks label to sell the products and inform the final customer about the services that the livestock provides.
Pau Costa Foundation coordinates and facilitates the initiative together with all the other stakeholders.

Degree of participation

Shepherds are engaged in the implementation phase of the project (grazing) and evalutaion (continuous feedback from them).Landowners are engaged in the planning phase (agreements to graze).
Catalan Fire and Rescue Service is involved in the planning phase (identifies the SMAs according to risk scenarios based on historical wildfire events and future projections).
The Catalan government (Climate Action Department) is involved in the planning phase (i.e. providing the funding for the payments for services and subsidies for grazing) and evaluation (certification of the grazing).
Local butchers and restaurants are involved in the implementation phase (selling of the products).
Pau Costa Foundation coordinates the stakeholders in all the different phases.

ADAPTIVE MANAGEMENT

Adaptive

There is no defined adaptive strategy. However, the project has been organically evolving in different phases and adapting to different contexts from its beginning. In that sense, during the initial year of the project, the focus was on promoting livestock products (i.e. increase its price as well as number of sellings). But in 2021, shepherds highlighted that the project was

not causing any impact on selling and products value. For this reason, the focus since then has been on payment for wilfire prevention service. The continuous communication between the different actors with the coordination team, has been fundamental to allow the project to be flexible to evolve and improve together with the different stakeholders to address potential problems.

Performance

Catalan Fire and Rescue Service is involved in the planning phase (identifies the SMAs according to risk scenarios based on historical wildfire events and future projections).

Funding

Fireflocks is a private initiative has received funding from several private and public bodies (e.g. competitive grants, awards, donations). The project started thanks to a private grant that allowed to implement the pilot with three shepherds (2016). Two years after (2020) the pandemic and the lack of budget stopped the project's growth, but the team was able to maintain the structure. Since 2022, the project expanded its implementation on new sites, growing of the project structure and incidence. At present, there is no structural funding supporting the initiative.
Complementary to the project's structure, in 2018, an agreement with the Catalan Climate Action Department linked the project with a subsidy for shepherds (ORDRE ARP/93/2018, 19 June) to graze SMAs. This agreement was key for the project to expand from three to 21 shepherds and for the livestock sector in general (receiving subsidies to graze strategic areas). While this funding is officially a subsidy and not a payment for a service, it was fundamental for the Fireflocks survivial to engage the shepherds in the project to graze SMAs, so they can receive the subsidy. Following the positive feedback from the different actors involved in the project, in 2023 the Fireflocks team strated collaborations with the public administration to develop an innovative payment system for the ecosystem services that the shepherds and the flocks provide.

WILDFIRE MANAGEMENT PHASES

Risk mitigation

From a risk mitigation perspective, Ramats de Foc promotes the management of SMA. These infrastructures are crucial in reducing wildfire behavior, such as flame length and rate of spread, thereby mitigating the impact of large wildfires. Planned at a landscape scale, SMAs create strategic zones that the fire service can utilize for operations during large wildfires. By maintaining these areas through controlled grazing, the initiative ensures that they remain effective barriers to wildfire progression, enhancing overall landscape resilience and providing critical support to firefighting efforts during large wildfire events.

Readiness

No readiness actions foreseen

Recovery

No recovery actions are foreseen.

6. SAFE VILLAGE AND PEOPLE PROGRAMS (Portugal)

Location: geographical boundaries and geographical and ecological features

All over the country (https://aldeiasseguras.pt/programa/).

What is it about? Main objectives

These are two programs created by Council of Ministers Resolution 157-A/2017 of October 27, in which the government established a set of measures aimed at introducing "a systemic reform in preventing and fighting forest fires, extending to other areas of protection and rescue". The "Safe Village" program is defined as a "Programme for the Protection of Population Clusters and Forest Protection" and is designed to establish "structural measures for the protection of people and property, and of buildings at the urban-forest interface, with the implementation and management of cluster protection zones and strategic infrastructures, identifying critical points and places of refuge". For its part, the "Safe People" program aims to promote "awareness-raising actions to prevent risky behaviour, self-protection measures and evacuation plan drills, in conjunction with local authorities".

Land use change tackled

No land use change tackled. The focus is on fuel management without necessarily change the land use. There is a recommendation to avoid trees with high flammability inside the home ignition zone. The aim is to prepare homes to withstand ember attacks and minimize the likelihood of flames or surface fire touching the home or the surrounding areas through the creation and maintenaince of fuel management strips.

It is advocated that the perimetric cleaning of the home surroundings and good management of a buffer strip are the most effective defence measures that the home owner can take against the arrival of a wildfire..

Predominant land use dynamics

These programs can be implemented in areas with different land use characterístics and dynamics. The establishment of the strips should be considered:

i) a length of 50 metres on land occupied by forest, scrub or natural pasture;

ii) a length not less than 10 metres in rural areas with other land use.

iii) in the buffer strip tree crowns must be at least 4 metres apart, except in the case of maritime pines and eucalyptus, where the minimum distance must be 10 metres. To avoid vertical continuity, trees over 8 metres tall must be pruned to at least 4 m above the ground. For trees less than 8 metres tall, pruning must be equivalent to 50% of their height;

' Trees and shrubs must be more than 5 metres from buildings and without their tops projecting over roofs.

Main entry point /common concern

The unprecedent wildfire disasters occurred in 2017 in mainland Portugal provoked 117 fatalities. These events and mainly the 67 fatalities in the Pedrógão Grande wildfire, occurred before the usual wildfire critical period, were deeply analysed and several recommendations to support a change in fire management policy were presented.The

Portuguese Government   wanted to act quickly and develop action to prevent and mitigate the effects of wildfires with immediate results. At the same time, the aim was to encourage greater citizen involvement by stimulating public participation and reinforcing the collective awareness that protection and safety are the responsibility of everyone and of all and for all - and this can only be achieved when accompanied by the adoption of appropriate measures to effective risk reduction for the settlements and populations living there.

ILA Dimensions

ILA Factors

MULTIFUNCTIONALITY

Multiple objectives

I. The protection of built-up areas through the management of buffer
zones and the creation of self-protection mechanisms;
II. Prevention of risky behaviour that could lead to fires starting;
III. Raising the population's awareness and notifications;
IV. Evacuation of built-up areas;
V. Preparation of shelters and places of refuge.

Practices undertaken

Practices are: 1- VILLAGES PROTECTION: citizens are responsible for establishing a defensible space. 2- PREVENT RISK BEHAVIOUR: actions undertaken by the national guard and municipalities to reduce fire outbreaks as a consequence of fire misuse. 3. SENSITIZATION AND ALERT TO POPULATION: when occuring is undertaken by nationla guard and municipalities. Generally, it is focused on fuel mnagamente and no information is provided beyind the fire weather index. 4- EVACUATION: this can be done with the support of national guard, firefighters and municipal staff. 5. DEFINITION OF A SHELTER /REFUGE: decided by municipal staff. 6. PROTECTION OF AGGLOMERATIONS is one of the measures established by the program with actions aimed at managing areas of protection zones located at the urban-forest interface, in order to reduce the possibility of buildings being affected by rural fires;

iT IS RECOMMENDED THE CONSTRUCTION TO THE PROTECTION OF fuel management strips around settlements and buildings, in order to
create discontinuity in the vegetation cover, increasing the level of safety for people and property and making the spaces located at the urban-forest interface (where they coexist) safer and more resilient and resistant to the action of fire. However no, support is done. The program just recomend what is already establish by law.

TRADE-OFFS

Accounting for synergies and trade-offs

The initiative does not consider trade-offs and synergies with other programs in place. Villages do nit have a plan beyond their evacuation plan. For instance, there is not measure to reactivate an agricultural buffer (with appropriate characteristics) that was common in the villages many years ago.

CROSS-SECTORAL

Multiple sector integration

The initiative involves at the strategic level the Portuguese Civil Protection responsible for the creation of the program and at operational level the municipalities and parish which must implement the programs . The engagement of the inhabitants of the villages is inexist or very reduced. The program adopts a top-down approach with no integration of local knowledge and need of the communities

Integrated management planning

The programs implementation is guided by a document with guidelines to support their implementation. It is based on the accomplishment of several measures without considering the characteristics of fire refuges. In the case of wildfires with high intensity advise people to go a refuge (an open space) increase their exposure to smoke, radiant heat, flames and spotting activity

PARTICIPATION

Transdisciplinary approach

The program namely considers Civil Protection ideas and does not involve other departments such as Forest management

Role

Municipalities select the villages to implement the programs and maintain them without assessing its effectiveness in changing the risk since they do not have the means to do this assessment. Municipalities do not select the areas to undertake buffer strips. Neighbours are contacted through awareness raising campaigns when municipalities are about to implement the programs. These campaigns are discontinued since people are not interested in them since they already kwno the legistation concerning fuel management surrounding their homes. Civil protection at the national level just created the programs and contabilize some metrics to assess the level of implementation of the programs.

Degree of participation

The people that would have interest/concern about the initiative, the neighbours, do not particiate. We would then only have municipalities and Civil Protection (who have the power to steer changes) involved, while the main interested actors, who are the citizens in high risk areas, are not engaged. The program is designed and implemented following a top-down approach where people's needs are not taken into account. The villages inhabitants

are not involved in the process. The sensitization campaigns in most of the villages just occurred in the moment of the programs implementation. It is very difficult to keep the interest of the people in the programs. There is no periodic evaluation of the project implementation. Besides the evacuation plan there is not a plan of strategic fuel management.

ADAPTIVE MANAGEMENT

Adaptive

There is no defined adaptive strategy and management. The changes of conditions are not considered.

Performance

The performance of the programs is measured by the general values of realization mentioned above (e.g., number of villages that have joined the programme, number of drills, etc) the quality of the implementation is not acessed. 2346 Villages Involved; 2194 Villages with a Local Security Officer; 991 Evacuation Plans; 532 Drills; 1510 Shelter Site; 1500 Places of Refuge. INFORMATION ON THE AMOUNT OF SURFACE CLEANED IS NOT AVAILABLE OR KNOWN

Funding

These programmes were created without a specific line of funding. ThePortuguese Civil Protection supported the implementation through providing signs to indicate places of shelter and refuge. Otherwise, the initiative does not have its own funds to support any action. The biomass reduction on the strips is the responsibility of the landowners. The municipalities are responsible of creating the strips in the urban areas and do not receive any funds from civil protection to implement the programs.

WILDFIRE MANAGEMENT PHASES

Risk mitigation

Reduce fire ignitions preventing risk behaviors; enhance people safety through evacuation, alerts, and the preparation of shelters and refuge areas; mitigate losses and damage reducing fuel load and enahnce self-protected measures. Although consir the fuel management any funding has been attributed to support it. There is not an approach to a strategic fuel management.

Readiness

Readiness actions are: evacuation plans, shelters and refuges, protect the houses in case of small fires

Recovery

The initiative does not consider recovery actions.

7. FIZ (Portugal)

Location: geographical boundaries and geographical and ecological features

Located in the Centre of the country, Pinhal Interior is one of the most fire prone Portuguese regions.

In Pinhal Interior, elevation ranges between 23 and 1418 meters a.m.s.l., and 46% of the surface has a slope of 25% or more. Landscape is dominated by forest and scrubland: 83% of the region's surface. Maritime pine (Pinus pinaster) and eucalyptus (Eucalyptus globulus) account for almost 2/3 of the region area. Agricultural areas occupy only 13% of the region, most of which are small-sized farms (67% are smaller than 5 ha).

Non-industrial private ownership is largely dominant in the region, with commons having a very small share of the whole area, although they can be more relevant in specific parishes. The initiative, which involves Forest Intervention Zones (FIZs) and more in particular those FIZ with an Integrated Landscape Management plan, takes place, therefore, mainly in non-industrial private owned land.

Forest Intervention Zones (FIZs), which have been established mainly between 2006 and 2011, occupy now 17% of the region: 774 km2 included in 26 FIZ. Since 2021 one third of this FIZ area (258 km2) is also under an approved Integrated Landscape Management Area (ILMA).

What is it about? Main objectives

The main objective of FIZs was the promotion of active forest management at a large multi-ownership level in order to reduce wildfire hazard in a context of small-scale ownership dominance. It takes place, therefore, mainly in non-industrial private owned land. It can been viewed as an innovative and demanding modality of multi-ownership collaboration. With the approval of an ILMA for some of the already existing FIZ, the main objective for multi-ownership management became the achievement of integrated landscape transformation. This territorial approach considers the relevance of forestry, farming and the ensemble of sectors, stakeholders and institutions that can play a role in wildfire reduction at local level.

Land use change tackled

One of the main actions achieved by the most active ZIFs was the involvement in the creation of a network of fuel breaks at the landscape level and its partial maintenance, often in articulation with municipalities and parish councils, in order to reduce the horizontal continuity of fuel load in a landscape dominated by continuous stands of Pine and Eucalyptus and, over all, to allow the entrance of firefighters equipment for suppression activities. The promotion of active forest management in individual stands to tackle with the trend of management abandonment, during last decades, in a considerable part of the more marginal stands, be it Pine or Eucalyptus (most Eucalyptus plantations took place in the 1980´s and are now in a third or more rotation) has been less successful. Besides those intervention, by promoting integrated landscape transformation ILMAs also aim at a global reduction of total forest area in order to tackle with the dynamics of progressive afforestation and scrubland increase since the 1950.

Predominant land use dynamics

Since the 1950 the region has witnessed a reduction in agricultural and pastoral land use (agricultural and pastoral cover) and increase in forest and scrubland.

Landscape is now dominated by forest and scrubland: 83% of the region's surface. Maritime pine (Pinus pinaster) and eucalyptus (Eucalyptus globulus) account for almost 2/3 of the region area. Agricultural areas occupy only 13% of the region, most of which are small-sized farms (67% are smaller than 5 ha).

Non-industrial private ownership is largely dominant in the region, with commons having a very small share of the whole area.

Main entry point /common concern

The years 2003, 2005 and 2017 stand out as those with the largest area burned in Portugal and in the region.

The creation of ZIFs in the Pinhal Interior region started soon after the approval of the ZIF legal frame in 2005. The establishment of a growing number of ZIFs from 2006 to 2011 in Pinhal Interior was triggered by several factors. First, non-industrial private owners had faced the consequences of those huge wildfires, with sometimes total devastation of their properties and were looking for a means of solving their management problems, namely the cost of interventions required to reduce wildfire hazard. Secondly, after being created under CAP requirements during the nineties, a large number of Forest Owners Associations was already operating on the ground and available to present themselves as ZIF management bodies.

ILMA were enacted after the 2017 wildfires. Their approval is expected to meant a shift of the policy approach to wildfire mitigation, from simple fuel reduction though forest management to integrated landscape transformation, and an expansion of the range of local entities that can lead the creation and management of the multi-ownership collaborative. It also led to a fundamental shift in eligible funds, which are expected now to support management costs, on a yearly basis, in addition to initial investment costs. The ILMA aims at overcoming the limitations of the FIZ, namely by admitting the need of earmarked public funding for landscape transformation and by allocating these funds to the most wildfire-vulnerable regions alone.

ILA Dimensions

ILA Factors

MULTIFUNCTIONALITY

Multiple objectives

The main objective of multi-ownership management under ZIF and ILMA plans is the promotion of active land management for the reduction of fuel loads, and increased availability of forest products (timber, bioenergy) and ecosystem services. With ILMA there is also an explicit emphasis on some agricultural products such as vine and olive growing.

Practices undertaken

One of the main actions achieved by the most active ZIFs was the active involvement in the creation of a network of fuel breaks and its partial maintenance, often in articulation with municipalities and parish councils. Under ILMA plans, deforestation and plantation or recuperation of old olive groves and vineyards is also considered.

TRADE-OFFS

Accounting for synergies and trade-offs

Synergies between wildfires reduction, timber production, water and biodiversity conservation are usually taken for granted in the elaboration of management plans and trade-offs are rarely considered. Besides the evaluation of synergies and trade-offs tend to ignore the management logics of the different actors. The bioenergy actors, for instance, have till now privileged the use of timber rather than the use of debris from fuels load interventions (pruning, thinning, and shrub clearing) due to transportation costs and efficacy.

CROSS-SECTORAL

Multiple sector integration

ILMA meant a shift of the policy approach to wildfire mitigation, from simple fuel reduction though forest management to integrated landscape transformation. This territorial approach considers the relevance of forestry, farming and the ensemble of sectors (tourism, energy, and environment), stakeholders and institutions that can play a role in wildfire reduction at local level.

Integrated management planning

The elaboration of the planning document is expected to reflect the integration of different sectors (forestry, farming, nature conservation, tourism) and stakeholders (forest owners, farmers, residents and their representatives; local government) involved in landscape transformation. The initiative encompasses several FIZ-ILMA in Pinhal Interior and, consequently, several management plans. Because different methodologies may have been followed for their elaboration, a more precise evaluation requires further analysis.

PARTICIPATION

Transdisciplinary approach

The initiative includes mainly actors from the policy (the National Forest Authority and the General Directorate for Territory are responsible for the management plans approval, respectively, for the FIZ and the ILMA) and practice (as a multi-ownership management collaboratives, landowners are essential actors along with Forest Owners Associations) related to wildfires.

Role

The initiative FIZ/ILMA in Pinhal interior includes both actors with power and/or interest on wildfire mitigation. Local Governments (mainly Municipal councils, and some Parish

councils) can be the proponents and the management body of the ILMA besides having competences on wildfire administration.

Degree of participation

Both the establishment of the FIZ and the ILMA and its management plan are formally accepted by a minimum of involved owners. However, the plan is initially drafted by technical staff rather than co-designed with owners.

ADAPTIVE MANAGEMENT

Adaptive

The shift of the rationale to wildfire mitigation, from simple fuel reduction though forest management (with silvicultural interventions such as shrub clearing and pruning, fuel breaks, and mosaic with different forest species and stand ages) to integrated landscape transformation (a ceiling for forest as % land cover and promotion of open areas, such as farmland) can be viewed as a response to the change in fire regimes and the informal evaluation of the effectiveness of the previous interventions.

Performance

A Systematic monitoring of performance has not been done under FIZ till now and is one of the main propositions presented to improve wildfire mitigation policy. Performance has been evaluated in an informal and non-comparative way.

Funding

While under FIZ alone, funding was available for only the first two years after the establishment and the support of initial establishment costs of the FIZ. The acknowledgement that the increase in management scale alone (under the multi-ownership collaborative comparing to individual non-collaborative management) is unable to overcome the cost constraints of active management (as assumed by FIZ policy) led to a fundamental shift in eligible funds, which are expected now to support management costs, on a yearly basis, in addition to initial investment costs, in ILMA.
The ILMA aims at overcoming the limitations of the FIZ, namely by admitting the need of earmarked public funding for landscape transformation and by allocating these funds to the most wildfire-vulnerable regions alone.

WILDFIRE MANAGEMENT PHASES

Risk mitigation

The goal and actions of FIZ/ILMA are centered on hazard mitigation, i.e. fuel management by (or on behalf of) land owners and land managers to reduce the amount and continuity of fuels, and not so much on adaptation, i.e. the creation of defensible or survivable space by homeowners to make their lives, livelihoods and property less vulnerable to wildfire damage.

Readiness

The initiative does not include readiness actions

Recovery

With ILMA it can be said that the initiative addresses recovery by considering not only pre disaster but mostly alternative scenarios, in opposition to what has been the dominant practice with FIZ.

8. CONTROLLED BURNING IN PASTORAL AREAS (Italy)

Location: geographical boundaries and geographical and ecological features

The central-western area of Sardinia (Italy), called "Planargia". This is an historical subregion characterized by herbaceous pastures and agroforestry areas with superficial rocks and stones, and with large portions of non-arable lands. In this area, pastoral fires to rejuvenate the pasture were usually carried out in September, shortly before the arrival of the first autumn rains: occasionally, these fires gave rise to large forest fires (Cabiddu et al. 2023). The core municipality where controlled burning in pastoral areas are applied is Suni, which is characterized by a high presence of agro-pastoral farms, and is located in the northern part of the province of Oristano.

What is it about? Main objectives

The main objective of this activity addresses the need of reducing uncontrolled burnings or even large, escaped wildfires, considering, at the same time, the agricultural and social use of fire in the area (improving the quality and palatability of grass species for livestock) and the temporal window needed for agro-pastoral purposes.

Land use change tackled

No land use change

Predominant land use dynamics

The area is characterised by agro-pastoral farms, where - back in the past - the cultural use of fire for agricultural and pastoral practices was very common to control weeds, remove woody shrubs, and facilitate livestock grazing. However, progressive pastoralism and agricultural decline in the 20th century, contributed to the progressive abandonment and a loss of technical knowledge of this practice, also due to the laws on fire regulation and fire prescriptions, mostly following a “zero-fire” policy. Before 2006, the use of fire was only allowed to private landowners during low fire risk periods, and to public land managers for controlling fuels in fuelbreaks or strategic areas but as an experimental tool.

Main entry point /common concern

This initiative was stimulated by the need of lowering the occurrence of large wildfires, which were typically “clandestinely” ignited and spread without any control at the end of the fire season, before the first fall rain events. Between 2002 and 2011, an average of 27 fires burned 340 hectares per year in the area, costing around 35,000 € per year for helicopter use (Cabiddu et al. 2023). The Sardinia Forest Service (CFVA), in conjunction with the local Municipality and the local community, decided to start this initiative in 2011.

ILA Dimensions

ILA Factors

MULTIFUNCTIONALITY

Multiple objectives

(a) Improving the quality and palatability of grass species for livestock; (b) decrease fuel loading/continuity to reduce the number of fires and mitigate potential losses; (c) train fire-fighting personnel in the technical use of fire; (d) resolve and prevent social conflicts related to the use and application of fire; (e) reduce the costs of extinguishing pastoral fires.

Practices undertaken

prescribed burning

TRADE-OFFS

Accounting for synergies and trade-offs

This initiatives considers synergies among agriculture and wildfire risk mitigation

CROSS-SECTORAL

Multiple sector integration

The main sector involved are agriculture and policy

Integrated management planning

The prescribed burning activities are described in a detailed planning of interventions, carried out by the CFVA. The controlled burning plan included: i) an analysis of the environmental constraints to consider for intervention design; ii) a socio-economic analysis and essential management interventions to limit smoke impact on communities; iii) a fire regime analysis to identify higher hazard and risk areas; iv) a detailed fuel analysis and estimate of potential fire behavior through field surveys and previous data.

PARTICIPATION

Transdisciplinary approach

Local stakeholders (agro-pastoral workers) propose areas of intervention through official request and in compliance with the regional regulations. The local public administration mediates the needs of local stakeholders and landmanager, thus bridging diffent aims and purposes. The National Research Council (CNR) is also involved supporting prescribed burning application with fuel moisture monitoring and fuel load assessment, as well as the collection of during- and post-fire metrics.

Role

Yes, this initiative includes several actors with different roles: 1) CFVA defines strategic zones where to carry out prescribed burning, checks, authorizes and plans the interventions to be executed in a given time-frame (generally late August – September), prepare the

prescribed burning plan, and conduct the burnings; 2) the local Municipality mediates the different interests, collects local stakeholders requests, and engage local stakeholders in meeting with CFVA; 3) local stakeholders fill specific authorization forms, propose area of intervention and support the CFVA during burnings; 4) CNR supports prescribed burning application with fuel moisture monitoring and fuel load assessment, as well as the collection of during- and post-fire metrics.

Degree of participation

Preparation: Local agro-pastoral workers fill specific authorization forms and ask the Forest Service to apply controlled fires in specific lands/properties; the local Municipality collects these requests and sends all documents to the Sardinia Forest Service; the Sardinia Forest Service checks, authorizes and plans the interventions to be executed in a given time-frame (generally late August – September);
Planning: the Sardinia Forest Service prepares and plan the controlled burning plan
Implementation: the Sardinia Forest Service carries out the controlled fires, sometime supported by local agro-pastoral workers; Research team at the National Research Council supports the controlled fires by measurements during the operations (e.g.: fuel load; fuel moisture; wind fields; tests in the use of foams or retardants; fire acceleration; etc.)
Monitoring: the Sardinia Forest Service analyses the impacts of the initiative (e.g.: area treated; area burned by wildfires; wildfire ignitions; team units occupied in wildfire suppression; etc.) to check if controlled fires are efficient in reducing wildfire ignition and spread

ADAPTIVE MANAGEMENT

Adaptive

The application of prescribed fire per se requires adaptation to weather conditions in order to identify the most favourable weather window. In some cases, adaptation was needed in order to take into account the farmer's needs, which required the application of prescribed fires for limiting the growing encroachment of Rubus ulmifolius (and/or other shrubs) in herbaceous pastures, that is for avoiding land use changes.

Performance

The results of prescribed burning applications are monitored. The results showed the positive effects of the application of controlled fires in terms of municipal reduction of areas burned, number of wildfires, hours of aerial interventions for firefighting. Regarding aspects of fire control, the prescribed fire program has significantly reduced the number of fires (-88%), the area burned (-91%), and the costs of aerial assets (-89%), comparing the periods 2011-2022 and 2002-2011. Furthermore, planned prescribed burning offered training on the prescribed fire technique, favoured the acquisition and improvement of teamwork techniques, and promoted a responsible approach to land management. At the same time, the application of low intensity and controlled fires allowed to safeguard forests from uncontrolled events and wildfires and to guarantee efficient renewals of herbs and pastures. As far as the agro-pastoral purposes are concerned, the combination of prescribed

burning before the rainy season allowed the growth of annual herbaceous vegetation palatable for livestock (grasses of the Hordeum and Lolium ssp. type and some legumes), thus guaranteeing a low-cost resource essential for extensive sheep farming (Cabiddu et al., 2023).

Funding

Funding for this initiative are largely stem from the Sardinia Forest Service, who provides the working hours to analyse the requests of controlled fire application and then perform in the field the activities. The personnel of the National Research Council contributes with budget coming from current and past projects (e.g.: EU Italy-France Maritime "MED-Star"; EU MSCA "Fire-Adapt"; EU H2020 "FirEUrisk").

WILDFIRE MANAGEMENT PHASES

Risk mitigation

The main objective of this initiative is to support local agro-pastoral workers in land management, by removing not very palatable herbs and renewing pastures using fire as cheap and controlled tool in stony and marginal zones, and to lower the occurrence of "clandestine" fires

Readiness

The initiative includes readiness actions to prepare local communities, which are informed about the execution of prescribed fires and how to behave in case of escaped fires or related issues. Moreover, fire and forest managers and services involved in the operations adopt specific safety plans (e.g.: definition of safe zones; escape routes; presence of lookouts) to limit the risk of escaped fires and optimize the controlled burning phases

Recovery

The initiative does not include recovery actions.

9. CARE (Collaborative Approaches for fire Resilient Ecosystems) (Portugal)

Location: geographical boundaries and geographical and ecological features

The study area is located in eastern-central Portugal, including three municipalities (Sabugal, Penamacor and Fundão) that cover 2,087 km2. It includes 2 AIGP ('Área Integrada de Gestão de Paisagem', meaning 'Integrated Landscape Management Areas') located in 2 mountainous reliefs of the Iberian Central System: AIGP' Serra da Gardunha' (in Fundão municipality) and AIGP' Terras do Lince' in Malcata mountain (in Sabugal and Penamacor municipalities). Those municipalities also include part of the Central' Meseta Iberica', divided by the Iberian Central System: the northern 'Meseta' (Sabugal northeast area) is a plateau marked by a highland landscape, with the main watercourse belonging to the Douro watershed, the southern 'Meseta' (Penamacor) is a peneplain surface where the watercourses flow into the Tagus river basin. The study area also includes geomorphological units known as 'Cova da Beira' (Northeast of Fundão and Southwest of Sabugal), a tectonic depression concerning the surrounding reliefs of the Estrela and Gardunha mountains also bellowing to Tegus basin. The study area is characterised by large and sparsely populated municipalities, linked to the rural abandonment of the last decades: Penamacor has the lower population density (8.5 inhab/km2) followed by Sabugal (13.7 inhab/km2) and Fundão (37.9 inhab/km2).

What is it about? Main objectives

CARE's goal is to assess the effectiveness of fire-risk mitigation actions implemented by local stakeholders through the analysis of their impact on fire-regulating services and other key ecosystem services (ES). Based on the obtained results, it also aims to promote a multi-actor discussion on synergies and trade-offs between those ES, to endorse the co-creation of nature-based solutions (NbS) linked to agrosilvopastoral mosaic, targeting fire-smart territories, to support decision-making process in the new Integrated Landscape Management Areas (AIGP) areas.

Land use change tackled

Land abandonment due to loss of profitability

Predominant land use dynamics

forests are the dominant land use, especially in Penamacor, covering more than half of the territory (between 1995 and 2918, passed from 50% to 60%); Fundão stabled around 44%. Both municipalities have a predominance of maritime pine (Pinus pinaster Aiton) and eucalyptus forests (Eucalyptus spp.). In Sabugal, forested areas slightly increased in the last decades (currently around 37%) and are dominated by the native black oak (Quercus pyrenaica Willd.) in pure stands or mixed in maritime pine. Regarding scrubland areas, Sabugal lost an extensive area and had 23% in 2018, either by conversion to forest or frequent fires in the southern part, leading to about 5% of the area with bare areas. Fundão and Penamacor also presented a reduction of shrublands in the last two decades, having nowadays around 12% and 10%, respectively. As for the agricultural area, in the last two decades, it had stable in Fundão (around 35%) and presented a slight decrease in Sabugal

(currently covers around 20%) and in Penamacor continues to decline, but less markedly over the past decade, and is currently at 17%. For pastures and agroforestry category, Sabugal presents a residually gaining over the last few decades (currently covers around 13%), Fundão has a stable tendency (almost 7% of the area) while Penamacor has also been losing area in these categories (currently has 11% of the area). While in Fundão and Penamacor, the agroforestry is dominated by cork oak (Quercus suber L.) and holm oak (Q. rotundifolia Lam.), in Sabugal dominates the Bocage landscape, a mosaic of mixed woodland (e.g. black oak) and pasture.

Main entry point /common concern

The Portuguese Landscape Transformation Programme (PLTP) is a national strategy for vulnerable forest territories with high fire risk, approved by the Ministers Council Resolution No. 49/2020, of 24 June. It is based on a participatory and collaborative vision to promote greater resilience in low-density rural territories and, simultaneously, encourage economic drivers linked to the agroforestry mosaic by valorising the natural and social capital. It also seeks to design the landscape and encourage and support local producers to adapt management practices to meet climate change challenges. The implementation of the PLTP in the medium and long term is carried out through two programmatic measures: the broader Landscape Planning and Management Programme (PRGP) and the local Integrated Landscape Management Areas (AIGP) (Decree-Law No. 28-A/2020). In our study area, the awareness of the high fire risk led the local authorities (municipalities) to propose 2 AIGPs: 1) AIGP Terras do Lince: With an area of 4.824ha was proposed jointly by Sabugal and Penamacor municipalities (Dispatch No. 7109-A/2021 of 16 July) and located in Malcata and Meimão parishes (located in Sabugal and Penamacor municipalities, respectively); 2) AIGP Serra da Gardunha: proposed by Fundão municipality (Dispatch No. 7109-A/2021) and has an area of 4.503ha that covers the central mountain area.

ILA Dimensions

ILA Factors

MULTIFUNCTIONALITY

Multiple objectives

>Assess fire-risk mitigation actions impact on key ES such as fire fire-regulating, climate-regulating, crop productivity, non-timber forest products, and recreation;
>Engage local stakeholders in the co-creation of nature-based solutions (NbS) linked to the agrosilvopastoral mosaic, targeting fire-smart territories;
>Support AIGP management entities in a collaborative decision-making process, considering ES trade-offs;

Practices undertaken

In the study area, the following practices have already been implemented: a primary network of fuel management strips and mosaics, prescribed burning, pastoralism with electric fences, and forestry management.

Practices planned to be implemented soon in the AIGP areas: converting scrubland, pine, and eucalyptus forests into agroforestry (dehesa), native forests, or spontaneous pastures; valuing existing agricultural and forest areas (pine, oak forests), also through the payment for ecosystem services.

TRADE-OFFS

Accounting for synergies and trade-offs

The initiative takes a participatory approach to discuss potential synergies and trade-offs between the 'fire-regulating ES' and other key ES. Using an ES bundle analysis as a tool for visualizing and communicating ES overlaps and mismatches could support an informed discussion between stakeholders to deliberate on fostering synergies and identify the acceptable trade-off between ES.

CROSS-SECTORAL

Multiple sector integration

The initiative involves multiple wildfires-related sectors: agriculture, forestry, pastoralism, local policy, culture, and tourism.

Integrated management planning

The Portuguese Landscape Transformation Programme (PLTP) has two programmatic measures that cover the study areas: 1.) the Regional Landscape Planning and Management Programme (PRGP) is a regional planning instrument for these rural territories aiming at regional landscape transformation to ensure the resilience and dynamisation of these territories; 2. The Local Integrated Landscape Management Areas (AIGP) are local-scale integrated territorial approaches that aim to foster landscape management to increase resilience to wildfires, natural capital valorisation and promotion of the rural economy. In the AIGP areas, the management model is defined by the Integrated Landscape Management Operations (OIGP) that identifies rural development and economic revitalisation interventions and details the extent and timing of agroforestry interventions, namely forestry, agriculture, and pastoralism to be implemented. CARE initiatives consider these integrated management planning strategies linked to the PLTP and integrate a collaborative strategy to co-create nature-based solutions (NbS) linked to agroforestry ecosystem services synergies and trade-offs.

PARTICIPATION

Transdisciplinary approach

The initiative considers multiple knowledge integration and includes diverse actors linked to fire prevention, agro-silvopastoral activities, local development, tourism, etc. The engaged actors come from policy (local and regional public administration, local politics, municipality technicians), practice (land managers and their local associations), science (forestry, pastoralism), and conservation (national/regional entities, local associations).

Role

The participation process started with a stakeholder mapping followed by an interest and influence analysis to identify who should be engaged. It includes actors with diverse roles in wildfire management, namely the ones with the power to take action (e.g. AIGPs management entities, the local politician) and those with active interest (e.g. land managers and their local associations).

Degree of participation

The CARE participatory approach is characterised by its co-creation process and the active involvement of multiple actors. It is structured into the following phases:
- 2021-23: Assessed local perception on key ES provided by agroforestry landscapes and mapped the selected ones (12ES), which were then discussed, validated and taken into consideration by stakeholders when they were asked to co-create macro-strategies to promote ES valorisation linked to the agroforestry mosaic;
- 2024-27: Creating a cross-border territorial co-design commission (including stakeholders from the neighbouring MOSAICO project) to define the progression of the participatory approach jointly, based on lessons-learn sharing and discussion on realistic and applicable NbS linked to existing rural dynamics;

ADAPTIVE MANAGEMENT

Adaptive

The Integrated Landscape Management Operation Plan (OIGP) details and articulates the operationalisation of AIGPs. Recently approved in one of the AIGPs covered by the study area, it includes a monitoring programme to track the implementation of actions to maintain and manage ecosystems. However, it does not specify any adaptive management strategy. CARE aims to support this evaluation and adaptation process by encouraging participation and collaborative creation of solutions based on joint analysis and discussion of the results obtained.

Performance

CARE aims to evaluate the effectiveness of fire-risk mitigation actions implemented by analysing their impact on fire-regulating services (based on wildfire spread/severity models) and other key ecosystem services. It also seeks to use metrics to evaluate the co-creation process's impact on local governance and decision-making based on simple data collection following participatory events. For example, the evaluation might consider whether the event was useful, whether it provided an important learning experience, or whether it influenced fire-management actions.

Funding

The AIGP management plan (OIGP) has 20 years of funding to support landscape transformation and maintenance, payment for ecosystem services, and monitoring activities. CARE is being supported by competitive projects, but the funding is available for a short-term period.

WILDFIRE MANAGEMENT PHASES

Risk mitigation

AIGPs are mostly focused on mitigating fire risk, namely through the conversion of more flammable land use classes (scrubland, pine forests, eucalyptus) native forests, agroforestry, orchards, and areas for annual crops or pastures. This also includes valorising existing agricultural areas, managing current forests, and payment for ecosystem services (e.g., of native forests with no associated economic income).

Readiness

The initiative does not include readiness actions.

Recovery

The initiative does not include recovery actions.

10. CITIZENS IN WUI (Greece)

Location: geographical boundaries and geographical and ecological features

he work focused on the island of Kythira, which lies south of Peloponnese, Greece, has an area of 277.28 km2 and a population of roughly 4000 people. The number of people on the island increases steeply in the summer with the return of Kythirians from Athens and the foreign countries to which they have emigrated, and with the addition of numerous tourists. Vegetation on the island, apart from small agricultural areas, consists mainly of evergreen Mediterranean shrubs and low xeric shrubs collectively called "phrygana". There are also limited areas of tall forest that cover 1.31% of the island and consist mainly of Pinus halepensis with an occasional mix of Eucalypt (Eucalyptus globulus).

What is it about? Main objectives

The project focused on the remote island of Kythira. It was prompted by a large wildfire that burned 8.91% (2,471 ha) of its total area. It was carried out by the Hellenic Society for the Protection of Nature in cooperation with the Institute of Mediterranean and Forest Ecosystems of the Hellenic Agricultural Organization DIMITRA. Its objective was to improve forest fire prevention on the island through better analysis of the fire problem, raising awareness of the population, and mitigation of risk to structures in cooperation with the population.

Land use change tackled

The project did not tackle land use changes. It only documented how changes in land use, population, and tourism have affected fire risk.

Predominant land use dynamics

Land abandonment as a result of population reduction (immigration), followed by shrub encroachment, with vegetation reaching the outskirts of villages, and tourist infrastructure development.

Main entry point /common concern

The main problem on Kythira regarding wildfires, is the availability of limited local firefighting resources. This, combined with difficulties in transportation of ground reinforcements by boat and delays in the arrival of aerial support due to distance, makes the vulnerability of settlements to fire a major concern. Thus, it is important to reduce the number of fire starts, and to improve self-protection of the population through appropriate education programs, including better preparation of structures. The latter was achieved through cooperation between scientists and residents.

ILA Dimensions

ILA Factors

MULTIFUNCTIONALITY

Multiple objectives

The work at Kythira integrated scientific analysis of the fire problem, that identified needs and high-risk locations, with the mobilization of residents and targeted work for reduction of vulnerability, in an innovative approach to fire prevention.

Practices undertaken

Fire potential assessment, with development of firk risk maps for authorities), Assessment of vulnerability of structures in three settlemenst with hep of volunteers, Land mng actions (fuel mng and reforestation), Citizen awareness campaigns

TRADE-OFFS

Accounting for synergies and trade-offs

Not explicitly accounted for

CROSS-SECTORAL

Multiple sector integration

The project blended many different sectors including: forestry, agriculture, tourism and civil protection.

Integrated management planning

No. The results that were delivered to the authorities (fire statistics analysis, forest fuel map, fire potential modelling, threat analysis across the island, etc.), form a good foundation for the development of a management plan by them.

PARTICIPATION

Transdisciplinary approach

Participation included the scientists of the two participating entities (a research Institute and an NGO), representatives from the municipality, the forest service, the fire service, the police, the local educators (elementary school and high school), students and the rest of the public. The later also included local shop owners who offered their premises for carrying-out workshops and other project activities.

Role

Local fire service, that had interest but not power to influence policies. Local teachers and citizens, with interest and no power. Local authorities had power and helped in mobilizing the citizens but did not explicitly uptake the recommendations.

Degree of participation

All the actors mentioned above (fire service, forest service, local authorities, tourism professionals, volunteers, teachers, students, residents, etc.) were informed, consulted and contributed to the development of the initiative through their active participation.

ADAPTIVE MANAGEMENT

Adaptive

After the end of the project there has been no strategy for adaptation to changing circumstances.

Performance

There has been no direct comprehensive evaluation of the results of the project. There are only indications of its success:
Satisfaction of citizens and students who asked for repetition of the activities, Satisfaction of the Fire Service with the outputs they received, Improved fire statistics (minimal area burned, fewer fire starts), No damages to homes.

Funding

This research was funded by the Green Fund of the Hellenic Ministry of Environment and Energy, in the frame of measure "Innovative actions with citizens", "Innovative Actions" axis of the "Natural Environment and Innovative Environmental Actions 2018" funding programme. The project started in April 2019 and was completed in October 2021, after an extension that was granted due to the Covid-19 pandemic.

WILDFIRE MANAGEMENT PHASES

Risk mitigation

The project focused and achieved: reduction of the number of fire starts through raising public awareness, Improvement in the resilience of structures following evaluation by house and delivery of assessment forms to more than 600 home owners. Better fire response planning by the Fire Service thanks to the fire analyses that were performed and delivered to them. Fuel management by volunteers at selected high-risk localities

Readiness

The project contributed to increased readiness of the people through educational workshops. Furthermore there were training videos on the subject that were created in the frame of the project

Recovery

The initiative does not address recovery actions
